# Predictive biomarkers for the efficacy of PARP inhibitors in ovarian cancer: an updated systematic review

**DOI:** 10.1038/s44276-025-00122-9

**Published:** 2025-03-11

**Authors:** Ying-Wen Wang, Isaac Allen, Gabriel Funingana, Marc Tischkowitz, Yvonne Walburga Joko-Fru

**Affiliations:** 1 Division of Gynaecologic Oncology, Department of Obstetrics and Gynaecology, Kaohsiung Chang Gung Memorial Hospital, Kaohsiung, Taiwan; 2 Department of Public Health and Primary Care, University of Cambridge, Cambridge, UK; 3 Department of Epidemiology, Harvard T. H. Chan School of Public Health, Boston, Massachusetts USA; 4 Department of Medical Oncology, Dana-Farber Cancer Institute, Boston, Massachusetts USA; 5 Department of Oncology, University of Cambridge, Cambridge, UK; 6 Department of Medical Genetics, National Institute for Health Research Cambridge Biomedical Research Centre, University of Cambridge, Cambridge, UK

## Abstract

**Background:**

PARP inhibitors are effective in treating ovarian cancer, especially for *BRCA1/2* pathogenic variant carriers and those with HRD (homologous recombination deficiency). Concerns over toxicity and costs have led to the search for predictive biomarkers. We present an updated systematic review, expanding on a previous ESMO review on PARP inhibitor biomarkers.

**Methods:**

Following ESMO’s 2020 review protocol, we extended our search to March 31, 2023, including PubMed and clinical trial data. We also reviewed the reference lists of review articles. We conducted a meta-analysis using a random-effects model to evaluate hazard ratios and assess the predictive potential of biomarkers and the effectiveness of PARP inhibitors in survival.

**Results:**

We found 375 articles, 103 of which were included after screening (62 primary research, 41 reviews). HRD remained the primary biomarker (95%), particularly *BRCA1/2* variants (77%). In the non-HRD category, six articles (10%) introduced innovative biomarkers, including ADP-ribosylation, *HOXA9* promoter methylation, patient-derived organoids, KELIM, and *SLFN11*.

**Discussion:**

Prospective assessment of real-time homologous recombination repair via nuclear RAD51 levels shows promise but needs validation. Emerging biomarkers like ADP-ribosylation, *HOXA9* promoter methylation, patient-derived organoids, KELIM, and *SLFN11* offer potential but require large-scale validation.

## Introduction

Ovarian cancer ranks as the fifth leading cause of cancer-related deaths among women in developed countries [1, 2], with epithelial ovarian cancer (EOC) accounting for 90% of cases [3]. Despite advances in therapy and surgery, EOC exhibits a high recurrence rate (up to 75% in advanced stages) [4]. Since 2011, survival was only marginally improved by the addition of bevacizumab to standard care [5, 6]. Over the past two decades, the overall 5-year survival rate for this condition has shown minimal improvement, with increases reported from around 31% to 34% in some regions [7].

Significant progress has been made with the advent of poly(ADP-ribose) polymerase (PARP) inhibitors, particularly in managing platinum-sensitive recurrent EOC [8, 9]. When used as maintenance therapy for patients responding to salvage chemotherapy, these inhibitors extend progression-free survival (PFS) by at least 5 months [10]. Notably, in the Phase 3 SOLO2 trial, a remarkable 19.1-month PFS benefit was reported for patients with *BRCA1/2* pathogenic variants versus placebo (*p* < 0.0001) [9]. Other Phase 3 trials, such as NOVA and ARIEL3, also demonstrated similar PARP inhibitor benefits in platinum-sensitive recurrent EOC with *BRCA1/2* pathogenic variants [8, 10]. Additionally, PARP inhibitors have shown efficacy as maintenance therapy in newly diagnosed, advanced-stage EOC patients [11, 12]. The efficacy of PARP inhibitors had also been affirmed in other cancer types, such as prostate cancer and breast cancer, particularly for genetically defined subgroups. In prostate cancer, the PROfound trial showed olaparib significantly improves outcomes in metastatic castration-resistant prostate cancer (mCRPC) patients with *BRCA1/2* and *ATM* pathogenic variants compared to hormone therapy [13], while TRITON3 highlighted rucaparib’s effectiveness in delaying disease progression for those with *BRCA1/2* pathogenic variants [14]. In breast cancer, OlympiAD demonstrated olaparib’s benefit in prolonging PFS for HER2-negative, *BRCA1/2*-mutated metastatic patients [15], and EMBRACA confirmed similar efficacy for talazoparib [16]. These findings underscore the value of PARP inhibitors in targeted treatment across cancer types.

The role of predictive biomarkers has been crucial in this context. The Phase 2 trial, Study 19, demonstrated the efficacy of olaparib for platinum-sensitive recurrent EOC, particularly in patients with *BRCA1/2* pathogenic variants, who experienced a significantly longer PFS [17]. ARIEL3 introduced tumour homologous recombination deficiency (HRD) as an additional predictive marker for PARP inhibitors, with significant PFS benefits for both *BRCA1/2* pathogenic variant carriers and patients with tumour HRD [10]. Other Phase 3 trials, including PAOLA-1, PRIMA, and VELIA, also demonstrated the benefits of PARP inhibitors in newly diagnosed advanced EOC patients with tumour HRD [12, 18]. *BRCA1/2* pathogenic variants and HRD are candidate predictive markers for PARP inhibitor effectiveness due to their roles in the homologous recombination repair (HRR) pathway. HRD cells employ non-homologous end joining (NHEJ) for DNA repair, resulting in less precise repair and increased DNA insertions and deletions [19].

Microhomology-mediated end joining (MMEJ) is another error-prone mechanism for repairing DNA double-strand breaks. This process involves aligning microhomologous sequences at the break ends and is often associated with deletions adjacent to the original break. DNA polymerase θ (Polθ) is crucial for MMEJ. In cells with HRD, these imperfect repair mechanisms further compromise genomic stability. This vulnerability makes PARP inhibitors particularly effective, as they disrupt single-strand DNA repair even more. This disruption results in the accumulation of DNA damage, leading to synthetic lethality and cell death [20].

The selection of EOC patients for PARP inhibitor treatment is increasingly based on the presence of *BRCA1/2* pathogenic variants and HRD status, with about 20% of EOC cases associated with *BRCA1/2* pathogenic variants [21]. One study examined tumour samples from over four thousand EOC patients to determine the frequency of tumour HRD and showed that 41.4% of patients across all histologic types exhibited tumour HRD. Among specific histologic types, serous carcinoma had the highest proportion of patients with tumour HRD at 42.4%. The prevalence of tumour HRD was 37.6% in endometrioid carcinoma, 13.6% in clear cell carcinoma, and 8.1% in mucinous carcinoma [22]. However, the clinical application of PARP inhibitors is not without challenges, including adverse events and concerns regarding cost-effectiveness. Patients treated with PARP inhibitors often experience a range of adverse events. Common non-haematologic side effects include nausea, fatigue, vomiting, diarrhoea, constipation, abdominal pain, and headache. Haematologic adverse events frequently observed include anaemia, neutropenia, and thrombocytopenia. Notably, approximately half of the patients may develop significant adverse events, which can impact the tolerability and management of treatment [8, 18, 23–28]. Moreover, the high cost of PARP inhibitors raises concerns regarding cost-effectiveness. Biomarkers such as germline/tumour *BRCA1/2* pathogenic variants or tumour HRD provide some patient stratification, potentially improving economic efficiency and patient safety [29, 30].

Establishing a universally accepted method or biomarker for patient stratification to maximise PARP inhibitor benefits is challenging. Approaches have focused on HRR genes like *BRCA1/2*, genomic instability markers such as genomic scars or mutational signatures, and functional assays such as RAD51 [31]. In view of the rapid advances in biomarkers and emerging non-HRD predictors of PARP inhibitor efficacy [32, 33] an updated systematic review is required.

## Materials and methods

**Fig. 1:**
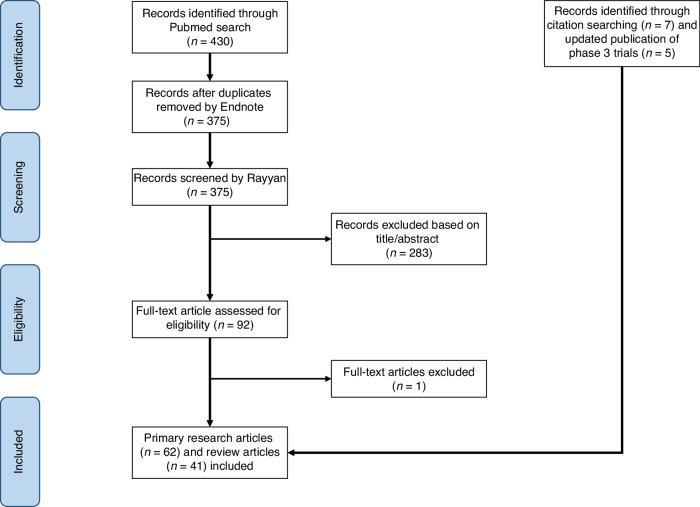
The PRISMA workflow.

This systematic review adheres to the PRISMA guidelines and follows the study protocol of the original ESMO systematic review [31]. The updated protocol for this systemic review was registered in PROSPERO with the study ID: CRD42023449959. For this updated review, we used the same search terms like the ESMO systematic review with updated search dates until March 31, 2023. We conducted a comprehensive search on PubMed and the clinical trial database (https://clinicaltrials.gov/), identifying 430 articles initially. After removing duplicates using Endnote (version X7.8), 375 articles remained. The search terms used for finding relevant articles are detailed in the Appendix Table 1. Figure 1 illustrates the PRISMA workflow.

We imported the 375 articles into Rayyan, a systematic review article screening platform. Two independent reviewers screened articles based on titles and abstracts using predefined inclusion and exclusion criteria. We included primary research articles or review articles which addressed ovarian cancer, assays or biomarkers, and PARP inhibitors. We excluded Phase 1 trials, studies unrelated to biomarkers (e.g., focused on the quality of life, pharmacokinetics, etc), and studies involving non-human subjects.

After screening, reviewer assessments were compared, and major discrepancies were resolved with input from a third reviewer. Minor discrepancies were resolved through discussion and full-text review. After resolving all discrepancies, we comprehensively reviewed the included articles. We assessed the level of evidence (LOE) by considering the study design and biomarker methodology [34]. We evaluated the quality of genomics-based predictive biomarkers using the Evaluation of Genomic Applications in Practice and Prevention (EGAPP) guidelines established by the US Centers for Disease Control and Prevention. This framework evaluates genomic assay’s analytic and clinical validity and utility [35]. Key information of articles included in this systematic review is presented in Appendix Table 2.

Our meta-analysis focused on Phase 2 and Phase 3 trials that provided hazard ratios (HR) of PFS for PARP inhibitors in ovarian cancer. This approach ensured consistency and comparability across studies. We observed that most biomarkers identified in the literature predict the survival benefit of PARP inhibitors. However, a subset of studies, specifically Phase 2 single-arm trials on newly diagnosed or recurrent EOC [36, 37], were excluded from the meta-analyses due to their lack of HR data on survival and divergent study designs, such as heterogeneity in patient enrolment or small sample sizes. The meta-analysis was conducted using the “metagen” function in RStudio. We anticipated the true effect sizes to vary between studies because of the heterogeneity in intervention effects and methodological approaches; therefore, the random-effects model was used to pool the effect sizes of the included studies. The DerSimonian-Laird estimator was utilised to assess between-study variance, and the I-squared was calculated to estimate the between-study heterogeneity. Detailed information on studies included in the meta-analyses can be found in the Appendix Table 3.

Previous reviews often presented germline and somatic *BRCA1/2* pathogenic variants as separate entities. However, due to their similar predictive capabilities and the widespread use of tumour *BRCA1/2* assays in clinical practice [12, 18, 38], there has been a noticeable shift towards amalgamating the results [8, 12, 18]. In prostate cancer, both germline and somatic *BRCA1/2* pathogenic variants similarly predict a favourable response to PARP inhibitors, as shown in pivotal trials like PROfound (olaparib) [13] and TRITON3 (rucaparib) [14]. In breast cancer, while germline and somatic *BRCA1/2* pathogenic variants also suggest potential sensitivity to PARP inhibitors, germline pathogenic variants are generally considered stronger predictors of efficacy. Major trials, such as OlympiAD [15] and EMBRACA [16], have primarily focused on patients with germline pathogenic variants. Although somatic *BRCA1/2* pathogenic variants are less common in breast cancer, they are potentially predictive but not as strongly linked to PARP inhibitor efficacy as germline pathogenic variants. Reflecting on this evolving approach, our review has been structured to provide a comprehensive assessment of the predictive capabilities of germline and somatic *BRCA1/2* pathogenic variants together.

## Results

The systematic review included 62 primary research articles, comprising 50 articles selected through screening in Rayyan and an additional 12 articles identified by screening the references of review articles. The predictive biomarkers proposed by these studies are summarised in Table 1.

**Table 1:** Summary of included articles by category of predictive biomarkers.

Category	Testing assay		Numbers of articles	Overall percentage
HRD test (59 articles)	HRR-related gene-level test (55 articles)	Germline or somatic *BRCA1/2* gene pathogenic variants (48 articles)	48	77%
		Pathogenic variants of HRR-related genes beyond *BRCA1/2* (18 articles)	15	24%
		HRR-related gene promoter methylation (7 articles)	7	11%
	Genomic scars and mutational signatures (33 articles)		33	53%
	Functional assay (7 articles)		7	11%
Non-HRD test (6 articles)	Other novel predictive biomarkers: ADP-Ribosylation, *HOXA9* promoter methylation, patient-derived organoids, KELIM, and *SLFN11* (6 articles)		6	10%

Studies may utilise more than one predictive biomarker.

The overall percentage is derived by dividing the number of articles by 62 and multiplying by 100%.

*HRR* homologous recombination repair, *HRD* homologous recombination deficiency, *ADP* adenosine diphosphate, *KELIM* CA-125 ELIMination Rate Constant K.

### HRR-related gene-level test

Of 62 articles in this review, 55 (89%) used assays to assess genes related to HRR as a primary method for detecting patients with HRD. The biomarkers suggested in the context of HRR-related genes can be grouped into three main categories: (1) germline or somatic *BRCA1/2* pathogenic variants, (2) pathogenic variants of HRR-related genes other than *BRCA1/2*, and (3) HRR-related gene promoter methylation. The majority of the studies were focused on detecting germline or somatic *BRCA1/2* pathogenic variants.

More than a hundred genes contribute to HRR, either through direct involvement or indirect mechanisms [39]. Central to the HRR pathway are the BRCA1 and BRCA2 proteins, which are critical because of their complex interactions with components like PALB2 and CHEK2 [40]. Additionally, significant roles are played by PALB2, ATM, ATR, CHEK1, CHEK2, RAD51, and genes linked to Fanconi anaemia [40]. BRCA1 assists in the initial processing of DNA double-strand breaks and interacts with PALB2. The combined action of BRCA1 and PALB2 aids BRCA2 in identifying processed DNA overhangs at the site of DNA double-strand break [40]. Once BRCA2 identifies the location of the single-strand DNA overhangs, it promotes the assembly of RAD51 recombinase, ensuring the progression of the subsequent HRR process [40].

### Germline or somatic *BRCA1/2* gene pathogenic variants

The primary and most widely employed predictive biomarker for assessing the effectiveness of PARP inhibitors is using assays to detect germline or somatic *BRCA1/2* pathogenic variants. Of the 62 studies reviewed, 77% utilised this particular assay as their predictive biomarker of choice. It is worth noting that these assays were predominantly commercial, with the Myriad MyChoice® CDx kit being the most prevalent at 46%, followed closely by Myriad BRACAnalysis® CDx at 40%, FoundationOne® CDx at 17%, and the BROCA Cancer Risk Panel at 6% of the studies that looked at *BRCA1/2*.

A key point of interest is that around 20% of EOC cases are associated with *BRCA1/2* pathogenic variants [21]. In the 2020 ESMO review, the results of major randomised clinical trials were summarised, clearly indicating that patients with *BRCA1/*2 pathogenic variants derive the greatest benefits from PARP inhibitors [31]. In this updated review, additional Phase 2/3 studies published after 2020 have been included. These additional data solidify the status of *BRCA1/2* pathogenic variants as an established predictive biomarker for assessing the efficacy of PARP inhibitors. The key findings of studies utilising *BRCA1/2* pathogenic variants to predict PARP inhibitor efficacy are illustrated in Appendix Table 4A.

### Pathogenic variants of HRR-related genes other than *BRCA1/2*

Of 55 primary research articles focusing on HRD predictability through HRR-related gene-level tests, 15 (27%) examined pathogenic variants in HRR-related genes other than *BRCA1/2* (Table 1). There is increasing interest in HRR genes beyond *BRCA1/2*, exemplified by studies involving *hMOB2* [41], *CCDC6* [42] and *EMSY* [43]. This was highlighted in the 2020 ESMO review, which underscored their varying impacts on synthetic lethality with PARP inhibitors [31]. Subsequent research has supported this concept. In ovarian cancer, pathogenic variants in *BRCA1, BRCA2, RAD51C, RAD51D*, and *PALB2* predict PARP inhibitor efficacy, but this has not been established for other HRR-related genes [44, 45].

Regarding the predictability of individual HRR-related genes and PARP inhibitor efficacy, the results of the ARIEL3 Phase 3 trial indicated that *RAD51C/D* pathogenic variants were predictive of the efficacy of rucaparib in platinum-sensitive recurrent EOC patients [45]. Other studies suggest associations between the expression of *EMSY*, *hMOB2*, *CCDC6-PP4*, and PARP inhibitor efficacy based on retrospective and in vitro findings that require further validation [41, 43]. On the other hand, some studies have explored HRD predictability using different HRR-related gene panels, but none have effectively predicted PARP inhibitor efficacy for EOC patients [46, 47]. These gene panels, designed to identify mutations within a select group of genes, do not adequately capture the multifaceted nature of HRD. Key findings from studies that investigated HRR-related genes other than *BRCA1/2* to predict the efficacy of PARP inhibitors are summarised in Appendix Table 4B.

When interpreting assay results detecting variants in *BRCA1/2* or other HRR-related genes, the impact on the HRR pathway is linked to their pathogenicity. Benign or likely benign variants are typically associated with a proficient HRR pathway, while likely pathogenic or pathogenic variants are linked to HRD. For variants classified as variants of unknown significance (VUS), the impact on the HRR pathway remains uncertain. While VUS rates specifically for *BRCA1/2* genes have decreased due to advances in classification and data collection, the overall VUS rate tends to increase with larger gene panels. This is because expanded panels include more genes, many of which are less well-characterised, leading to a higher likelihood of identifying VUS [48]. Kurian et al. investigated trends in genetic testing among women with breast or ovarian cancer and found that the VUS rate increased from 8.1% in 2013 to 28.3% in 2017 with the use of broader HRR-related gene panels [49].

### HRR-related gene promoter methylation

Genetic alterations and epigenetic changes, such as DNA methylation, can influence HRR capacity by modulating gene regulatory elements. DNA methylation can either silence (hypermethylation) or activate (hypomethylation) genes [50]. The ESMO review emphasised the need for stronger evidence to validate HRR-related gene promoter methylation as a predictor of PARP inhibitor efficacy due to doubts about earlier research reliability [31]. In this updated review of 55 articles using gene-level HRD assays, only 7 (13%) addressed HRR-related gene promoter methylation.

While *BRCA1* promoter methylation is associated with HRD and is present in a subset of high-grade serous ovarian cancer (HGSOC) cases, findings from the TCGA study [21], indicate that *BRCA1* promoter methylation was not highlighted as an independent prognostic marker for patient outcomes. This aligns with the observation that while *BRCA1* promoter methylation contributes to the molecular characterisation of HGSOC, its role as a prognostic indicator remains uncertain.

Recent evidence has highlighted that an additional 10% of EOC patients exhibit “BRCAness”, which refers to tumours that share molecular features with tumours possessing pathogenic variants of *BRCA1/2*, such as those identified through HRR-related gene promoter methylation, beyond what genetic testing alone can reveal [51]. Further analysis of the Phase 2 ARIEL2 trial showed that *BRCA1* promoter hypermethylation is associated with improved PFS in recurrent EOC patients with wild-type *BRCA1/2* (*p* = 0.01) [52]. Some studies suggest extended overall survival and heightened sensitivity to platinum and PARP inhibitors among patients with *BRCA1/2* promoter methylation [53]. These findings are retrospective and preclinical and thus require validation in larger prospective studies [53–55]. In summary, even with new studies exploring HRR-related gene promoter methylation as a predictive biomarker for PARP inhibitor efficacy, its predictability remains inconclusive. The key findings of reviewed studies utilising HRR-related gene promoter methylation to predict the efficacy of PARP inhibitors are summarised in Appendix Table 4C.

### Genomic scars and mutational signatures

#### Genomic scars

Genomic scars, or mutational signatures resulting from HRD, provide essential markers for HRD detection. These signatures encompass various DNA copy number alterations, including Loss of Heterozygosity (LOH), Telomeric Allelic Imbalance (TAI), and Large-Scale State Transitions (LST).

LOH, a genomic phenotype, often emerges following HRD [56]. Abkevich et al. classified LOH into small, intermediate, or large categories, observing a significantly higher prevalence of intermediate LOH regions in tumours with *BRCA1* or *BRCA2* deficiency (*p* = 10^−^^11^) [56]. TAI refers to the unequal distribution of alleles at chromosome ends, which is also indicative of HRD and is associated with a positive response to DNA-damaging agents like cisplatin [57]. This relationship was confirmed in breast cancer patients, with cisplatin-sensitive individuals showing higher TAI levels compared to resistant individuals (median TAI: 24 vs. 17.5, *p* = 0.047) [57]. Tumorigenesis often leads to increased DNA breaks and genomic fragmentation, resulting in LST, which are large chromosome rearrangements characterised by breaks spanning at least 10 Mb between neighbouring regions, excluding the centromere. LST is a marker of genomic instability and indicates *BRCA1/2* deficiency [58]. In their study, Popova et al. examined breast cancer cells and found that an elevated number of LST events effectively distinguished cells with defective *BRCA1/2* genes from those with proficient *BRCA1/2* genes (*p* < 10^−^^6^) [58].

The clinical validity and utility of genomic scar assays have been affirmed in the ESMO review, summarising their predictive value in Phase 3 studies like ARIEL3, PRIMA, VELIA, and PAOLA-1. The most commonly utilised assays are MyChoice® CDx and FoundationOne® CDx, which have evolved from the concept of genomic scars mentioned earlier [31]. In this updated review, out of 62 primary research articles, 33 (53%) used genomic scars or mutational signatures to detect HRD in cancer cells. Most articles focused on genomic scars, with two utilising the mutational signature approach [46, 59]. Beyond the Phase 3 studies covered in the ESMO review, the predictability of genomic scar assays has received additional support from subsequent clinical trials, such as OPINION [60], LIGHT [36], MITO16A [61], OVARIO [37] and ATHENA-MONO [62]. The key findings of the included studies that utilise genomic scars to predict the efficacy of PARP inhibitors are summarised in Appendix Table 4D.

When interpreting the results of genomic scar assays, the uncertain predictability of these assays for the survival benefits of PARP inhibitors is worth considering. The HRD score, primarily derived from FoundationOne® CDx and Myriad MyChoice® CDx, offers an approach to quantify genomic scars within the cancer genome and predict the efficacy of DNA-damaging agents in ovarian cancer. As introduced by Telli et al., the HRD score compiles three genomic scars—LOH, TAI, and LST—into an unweighted sum. An HRD score of 42 or higher indicates increased sensitivity to DNA-damaging agents, such as platinum and PARP inhibitors [63]. However, clinical trials have elucidated the complex nature of predicting treatment outcomes using HRD scores. For example, the Phase 3 PRIMA trial, focusing on niraparib as maintenance therapy for newly diagnosed advanced EOC, demonstrated survival benefits of using niraparib for patients with proficient HRR pathways (HRD score < 42), resulting in a hazard ratio of 0.68 with a 95% CI of 0.49–0.94 compared to the control group [38]. However, long-term follow-up data from PRIMA, published recently, indicated no difference in OS between the treatment and control arms (HR 0.93, 95% CI: 0.69–1.26) after a median follow-up of 73.9 months [64]. The Phase 3 ATHENA-MONO trial supported the effectiveness of rucaparib monotherapy as a first-line maintenance option across various subgroups of newly diagnosed advanced EOC, including those with different levels of LOH and *BRCA1/2* wild-type status. Specifically, for patients with *BRCA1/2* wild-type and low LOH, the hazard ratio of rucaparib compared to the control was 0.65 (95% CI: 0.45 to 0.95), for those with intermediate LOH, it was 0.39 (95% CI: 0.20 to 0.78), and for those with high LOH, it was 0.58 (95% CI: 0.33 to 1.01) [62]. Furthermore, the Phase 3 ARIEL3 trial for platinum-sensitive recurrent EOC revealed that patients without germline *BRCA1/2* pathogenic variants and with low LOH also benefited from rucaparib, with a hazard ratio of 0.58 (95% CI: 0.40–0.85, *p*-value = 0.0049) [10]. This finding indicates that even those with wild-type *BRCA1/2* genes and low HRD scores can derive survival advantages from PARP inhibitors compared to the control group.

### Mutational signatures

Genomic scars, which are primarily derived from chromosome microarrays, provide a broad view of genomic instability by detecting large-scale DNA alterations. In contrast, mutational signatures offer a more detailed snapshot of the genome. These signatures detect pathogenic variants associated with tumorigenesis and encompass single nucleotide substitutions, small insertions/deletions, and large-scale structural rearrangements through whole exome or whole genome sequencing. Among single nucleotide substitution mutational signatures, researchers have proposed 21 major types [65]. Signature 3, for example, found in 14.4% of pan-cancer samples, is mainly associated with *BRCA1/2* pathogenic variants and pathogenic variants in other HRR-related genes, making it a potential biomarker for HRD [66]. However, it is important to note that various HRR-related genes have different impacts on mutational signatures. *BRCA1/2* pathogenic variants predominantly contribute to signature 3 and, to a lesser extent, signature 8 of base substitution [66]. HRD resulting from *BRCA1/2, RAD51C*, or *PALB2* inactivation often exhibits dominance of signature 3, while HRD due to *CHEK2* or *ATM* pathogenic variants may not [67].

To address the complexity of HRR-related genes and limitations in individual mutational signatures for predicting PARP inhibitor efficacy, HRDetect integrates multiple features of mutational signatures. These features include base-substitution signatures 3 and 8, rearrangement signatures with increased large deletions (>3 bp) and microhomology at junctions, rearrangement signature 5, specific copy number variations, and LOH. HRDetect excels with an AUC of 0.98 and 98.7% sensitivity for *BRCA1/2* deficiency and HRD identification (cutoff: 0.7). Its performance is optimal with whole genome sequencing and diminishes with exome or targeted panel sequencing [68].

The ESMO review found no significant evidence to confirm the reliability of mutational signatures from whole genome sequencing as predictors of PARP inhibitor effectiveness. In this updated review, three subsequent studies (Färkkilä et al. along with two preclinical studies [46, 54, 55]) used mutational signatures or HRDetect as biomarkers for PARP inhibitor efficacy in EOC. In the TOPACIO trial, Färkkilä et al. found that only mutational signature 3 significantly correlated with a longer median PFS (5.0 months vs. 2.2 months, *p* = 0.0005) in recurrent EOC patients [46]. However, the two preclinical studies did not yield conclusive results for HRDetect and mutational signature 3 [54, 55]. A summary of key findings from studies using mutational signatures to predict PARP inhibitor efficacy is provided in Appendix Table 4D.

Interpreting genomic scars and mutational signatures has its limitations as mutational signatures can vary based on the duration and extent of mutagen exposure, reflecting the evolving complexity of tumorigenesis. Additionally, mutational signatures can emerge during the precancerous stage. Genomic scar assays offer a “snapshot” of the cancer genome, potentially misrepresenting the current tumour status [69]. For instance, cells can regain HRR capability through *BRCA1/2* mutation reversion and develop resistance to PARP inhibitors, even when their genomic scar score indicates HRD [69].

### Functional assays for detecting HRD

Functional assays for HRD provide real-time insights into the capacity of HRR, offering advantages over “genomic scars” and “mutational signatures.” Unlike these other methods, which reflect accumulated historical damage and may not indicate the current state of HRR, functional assays assess the active repair capacity of tumours. This dynamic assessment allows for more accurate identification of HRD-positive tumours and can detect cases where HRR proficiency has been restored through mechanisms like reversion mutations. Additionally, functional assays may offer better predictive power for treatment response by measuring actual repair activity, enhancing their utility for guiding therapy decisions.

Among these assays, the RAD51 assay is a prominent method for assessing HRD. This assay evaluates the role of RAD51 in repairing DNA double-strand breaks through the HRR pathway, which involves essential components like BRCA1, BRCA2, and PALB2. RAD51 plays a crucial role in assembling recombinase on single-strand DNA overhangs, a critical step in HRR [40]. In 2010, Graeser et al. demonstrated its predictive value in breast cancer patients undergoing neoadjuvant chemotherapy, showing that tumours with a complete pathological response had lower RAD51 scores compared to non-responders (median RAD51 score: 2.6% vs 44%, *p* = 0.028) [70]. Initially validated in preclinical studies and smaller patient groups, the assay is now gaining clinical recognition for HRD diagnosis and predicting PARP inhibitor response.

The ESMO review found insufficient evidence to support the clinical validity of RAD51 functional assays in predicting PARP inhibitor responses. In this updated review of 62 studies, 7 articles (11%) utilised the RAD51 assay. Among these, four clinical studies provided valuable insights: Two clinical studies indicated improved survival for patients with low RAD51 levels [71, 72]. A post-hoc analysis of the MITO16A Phase 4 study suggested comparable predictability between the RAD51 assay and the Myriad MyChoice® CDx assay in predicting the efficacy of platinum-based chemotherapy [61]. However, in the TOPACIO Phase 2 trial, RAD51 did not consistently predict treatment response in platinum-resistant recurrent EOC patients receiving niraparib and pembrolizumab [46]. Apart from these clinical findings, additional case series and preclinical studies [54, 55, 73] have also contributed to evidence regarding the predictive value of RAD51.

Assessing nuclear RAD51 recombinase levels provides a functional evaluation of the HRR pathway. However, the absence of RAD51 recombinase formation does not always indicate defects in the later stages of HRR [74]. Downstream impairments that can affect HRR include defects in RAD54, which disrupt the resolution of repair intermediates [75], and alterations in endonucleases such as MUS81-EME1, which impact the final resolution of DNA structures [76]. These defects can result in partial, error-prone repair and contribute to genomic instability. Key findings from studies using the RAD51 functional assay to predict PARP inhibitor efficacy are summarised in Appendix Table 4E.

### Other biomarkers

Six articles were included that explore alternative biomarkers that are not directly linked to the HRR pathway but hold promise in predicting the effectiveness of PARP inhibitors. A concise overview of the key findings from studies employing these non-HRR pathway biomarkers to predict PARP inhibitor efficacy is provided in Appendix Table 4F.

### ADP-ribosylation

One study introduces ADP-ribosylation as a potential predictor for PARP inhibitor efficacy. ADP-ribosylation, a posttranslational modification affecting protein function, has been associated with cellular processes such as stress response and metabolism [77]. Conrad et al. analysed tumour samples from high-grade serous carcinoma (HGSC) patients and identified distinct molecular phenotypes based on ADP-ribosylation levels. These patterns correlated with RNA expression profiles and clinical outcomes. Elevated PARP-1 levels were also found to drive increased ADP-ribosylation in ovarian cancers, with olaparib sensitivity relating to ADP-ribosylation levels [32].

### Methylation status of *HOXA9* promoter

The Phase 2 study conducted by Rusan et al. suggests that changes in *HOXA9* promoter methylation may serve as a predictor of PARP inhibitor efficacy. *HOXA9*, a member of the HOX gene family, plays a crucial role in solid tumour development [78]. Rusan et al. investigated the response to veliparib in patients with platinum-resistant recurrent *BRCA1/2*-mutated EOC, monitoring *HOXA9* promoter methylation in circulating tumour DNA. While initial results did not reach statistical significance, post-veliparib treatment changes in *HOXA9* methylation correlated with survival outcomes, suggesting its potential as a predictive biomarker. However, these findings are preliminary and should be interpreted with caution, as further investigation is necessary to confirm their clinical relevance [78].

### Patient-derived organoids

Two studies explored the use of patient-derived organoids to assess PARP inhibitor sensitivity in ovarian cancer patients. Organoids provide a more realistic representation of organ complexity compared to traditional in vitro cell line studies. Tao et al. demonstrated the feasibility of using ovarian cancer organoids to predict PARP inhibitor responses in a small patient cohort [73], while Sheta et al. generated 3D organoids from cancer cells of HGSC patients and identified distinct gene expression differences between PARP-sensitive and PARP-resistant organoids [79].

### KELIM (CA-125 ELIMination Rate Constant K)

One study proposes using the KELIM score, initially designed for predicting chemosensitivity in ovarian cancer, as a predictor of PARP inhibitor efficacy. Based on the CA-125 decline over the initial cycles of platinum-based chemotherapy, the KELIM score has shown reliability in assessing chemosensitivity. An integrated analysis of two Phase 2 trials evaluating rucaparib’s efficacy in recurrent EOC demonstrated that patients with a favourable KELIM score experienced a significant reduction in the risk of disease progression or death when treated with rucaparib, with a HR of 0.67 (95% CI: 0.50–0.91) compared to those with an unfavourable KELIM score. These findings suggest that the KELIM score may serve as a valuable tool in identifying patients who are more likely to benefit from rucaparib treatment [33].

### Schlafen 11 (SLFN11)

*SLFN11*, known for inducing irreversible replication block, is emerging as a predictive biomarker in various cancer types, including ovarian cancer. A retrospective analysis showed that patients with high SLFN11 expression had a median OS of 80 months (95% CI: 55–105) compared to 49 months (95% CI: 38–60) for those with low expression (*p* = 0.016), which suggested a positive correlation between *SLFN11* levels and the effectiveness of DNA-damaging agents such as platinum and PARP inhibitors [80]. The other retrospective study demonstrated that higher *SLFN11* expression was associated with platinum sensitivity and extended PFS [81]. These findings underscore *SLFN11*’s potential as a predictive biomarker for guiding treatment strategies.

In Table 2, we present all the new or updated papers in comparison with the 2020 ESMO systematic review.

**Table 2:** Comparison of articles in the 2020 ESMO review and in this updated review

Cancer type	Study design	Included articles											
**Ovary (PARP)**	Study 19, Study 41, Study 42 (Phase 2)	**Ledermann et al.** [84]	**Ledermann et al.** [17]	**Domchek et al.** [85]		**Ledermann et al.** [86]	**Matulonis et al.** [87]	**Dougherty et al.** [88]	**Lheureux et al.** [89]	**Hodgson et al.** [90]		**Friedlander et al.** [91]	
	NOVA (Phase 3)	**Mirza et al.** [8]	*Del Campo* et al. [92]										
	AVANOVA2 (Phase 2)	**Mirza et al.** [93]											
	Study 10 and ARIEL2 (Phase 2)	**Swisher et al.** [94]	**Kondrashova et al.** [95]	*Kristeleit* et al. [96]		*Swisher* et al. [52]	*Colomban* et al. [33]^*a*^ *(KELIM)*	*Kristeleit* et al. [97]					
	ARIEL3 (Phase 3)	**Coleman et al.** [10]	*Clamp* et al. [98]	*Oaknin* et al. [99]		*O’Malley* et al. [45]							
	SOLO2 (Phase **3)**	**Pujade-Lauraine et al.** [9]	*Poveda* et al. [25]	*Frenel* et al. [100]		*Liu* et al. [101]							
	SOLO1 (Phase 3)	**Moore et al.** [11]	*Banerjee* et al. [26]	*Wu* et al. [82]		*DiSilvestro* et al. [102]							
	VELIA	Coleman et al. [18]	*Aghajanian* et al. [103]	*Swisher* et al. [104]		*Mizuno* et al. [105]							
	QUADRA (Phase 2)	**Moore et al.** [106]	*Okamoto* et al. [107]										
	PAOLA-1 (Phase 3)	Ray-Coquard et al. [12]	*Fujiwara* et al. [108]	*Harter* et al. [109]		*Pujade-Lauraine* et al. [47]	*Callens* et al. [110]	*Loverix* et al. [111]	*Christinat* et al. [112]	*Ray-Coquard* et al. [23]			
	PRIMA (Phase 3)	González-Martín et al. [38]	*González-Martín* et al. [38]										
	TOPACIO (Phase 2)	**Konstantinopoulos et al.** [113]	*Färkkilä* et al. [46]										
	Other clinical trials	**Gelmon et al.** [114] **(Phase 2)**	**Kummar et al.** [115]	**Oza et al.** [116] **(Phase 2)**		**Drew et al.** [117]	**Liu et al.** [118] **(Phase 2)**	*Rusan* et al. [78] *(Phase 2, HOXA9 promoter methylation)*^*a*^	*Lee* et al. [119] *(AMBITION, Phase 2)*	*Chen* et al. [120] *(NCI-MPACT, Phase 2)*		*Penson* et al. [27] *(SOLO3, Phase 3)*	
		*Poveda* et al. [121] *(OPINION, Phase 3)*	*Poveda* et al. [60] *(OPINION, Phase 3)*	*Wu* et al. [82] *(NORA, Phase 3)*		*Cadoo* et al. [36] *(LIGHT, Phase 2)*	*Hardesty* et al. [37] *(OVARIO, Phase 2)*	*Kristeleit* et al. [83] *(ARIEL4, Phase 3)*	*Liu* et al. [122] *(NRG-GY004, Phase 3)*	*Monk* et al. [62] *(ATHENA-MONO, Phase 3)*		*Liu* et al. [123]	
	Other relevant studies	**Graeser et al.** [70]	**Lips et al.** [124]	**Mukhopadhyay et al.** [125]		**Shah et al.** [126]	**Lee et al.** [127]	**O’Donnell et al.** [128]	**Zhao et al.** [129]	**Cruz et al.** [130]		**Castroviejo-Bermejo et al.** [131]	
		**Kondrashova et al.** [132]	**Funnell et al.** [133]	*Sahnane* et al. [53]		*Coelho* et al. [134]	*Guffanti* et al. [54]	*Pellegrino* et al. [55]	*Gundogdu* et al. [41]	*Morra* et al. [42]		*Agarwal* et al. [135]	
		*Conrad* et al. [32]^a^ *(ADP-Ribosylation)*	*Sheta* et al. [79]^*a*^ *(3D organoid)*	*Tao* et al. [73]^*a*^ *(3D organoid)*									
**Ovary-Platinum**	Clinical trials	**Norquist et al.** [136] **(GOG-218 (Phase 3))**	**Stronach et al.** [137] **(SCOTROC4, Phase 3)**	[61] (MITO16A, Phase 4)									
	Other relevant studies	**Swisher et al.** [138]	**Alsop et al.** [139]	**Pennington et al.** [140]		**Bernards et al.** [141]	*Wen* et al. [142]	*Costa* et al. [143]	*Hollis* et al. [43]		*Wijk* et al. [144]		*Winkler* et al. [81]
**Other cancer type**	Clinical trials	**Mateo et al.** [145] **(TOPARP, Phase 2, prostate)**	**Goodall et al.** [146] **(TOPARP, Phase 2, prostate)**	**Roviello et al.** [147] **(OLTRE, Phase 2, breast)**		*Blanc-Duran* et al., [72] *(CHIVA, Phase 2)*							
	Other relevant studies	**Quigley et al.** [148]											
**The development of assays**	**Mukhopadhyay et al.** [149] **(RAD51)**	**Popova et al**. [58] (LST)	**Birkbak et al**. [57] **(TAI)**	**Abkevich et al.** [56] **(LOH)**		**Frampton et al.** [150] **(FoundationOne)**	**Zhang et al.** [151] **(Genomic scars)**	**Telli et al.** [63] **(MyChoice)**	**Davies et al.** [68] **(HRDetect)**				
**Others**	**Bryant et al.** [152]	**Farmer et al.** [153]	**Edwards et al.** [154]	**Sakai et al.** [155]		**Wang et al.** [156]	**Nomura et al.** [157]	**Maxwell et al.** [158]	**Prieske et al.** [159]	**Wang et al.** [160]		**Deniz et al.** [161]	
	**Weigelt et al.** [162]	*Kang* et al. [163]	*Aref-Eshghi* et al. [51]										

In the table, bold entries denote articles in the original ESMO review, entries in italic represent articles in this updated review, and underlined table entries indicate articles in both reviews. Articles utilising the same study cohort are positioned within the same row. For instance, Mirza et al. reported the primary findings in the NOVA trial in 2016, while Del Campo et al. conducted a post-hoc analysis in 2019. Since both studies employ the same study cohort, they are aligned in the same row. Other individual trials or studies have been grouped within rows due to study design similarities.

*HRR* homologous recombination repair, *HRD* homologous recombination deficiency, *ADP* adenosine diphosphate, *KELIM* CA-125 ELIMination Rate Constant K, *3D* three-dimensional, *LOH* loss of heterozygosity, *TAI* telomeric allelic imbalance, *LST* large-scale state transition.

^a^Articles that utilise other novel predictive biomarkers (not related to homologous recombination deficiency).

### Results of the meta-analyses

**Fig. 2:**
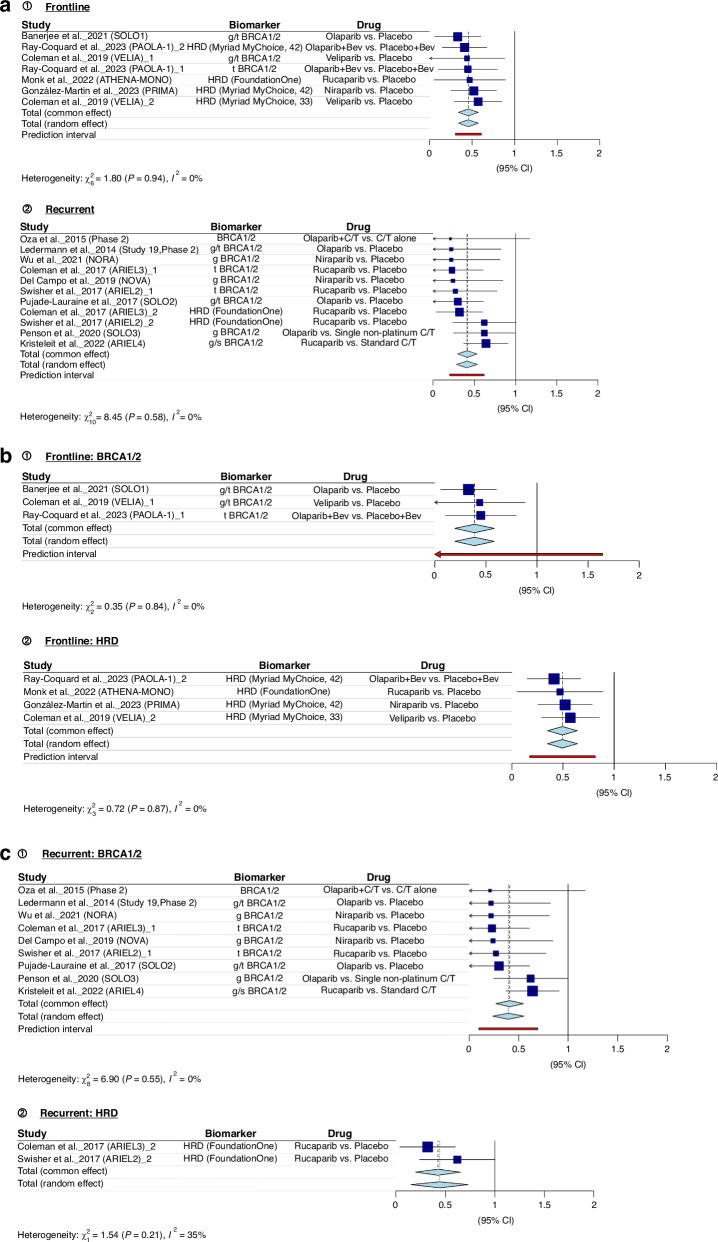
Meta-analysis: predictive biomarkers in PARP inhibitor efficacy. **a** *BRCA1/2* pathogenic variants and tumour HRD as predictors of PARP Inhibitor efficacy across treatment settings. **b** *BRCA1/2* pathogenic variants and HRD in frontline treatment. **c** *BRCA1/2* pathogenic variants and HRD in recurrent treatment.

Figure 2 presents the findings of our meta-analyses. Figure 2a consistently demonstrates that both *BRCA1/2* pathogenic variants and tumour HRD serve as robust indicators of the effectiveness of PARP inhibitors. In newly diagnosed advanced EOC (frontline) and recurrent EOC, the combined hazard ratio (pooled HR) falls below 1, indicating a substantial treatment advantage (pooled HR = 0.4552; 95% CI: 0.3387; 0.5717). The examination of heterogeneity across studies shows minimal variation (*I*² = 0.0%, *p* = 0.9375), affirming the reliability of these results. Figure 2b and c provide separate meta-analyses for *BRCA1/2* and HRD in the context of frontline and recurrent treatments, respectively. Details of all articles included in the meta-analysis are presented in Appendix Table 3.

## Discussion

This review extends the ESMO search period from September 25, 2019, to March 31, 2023, providing updated evidence. It includes Phase 2 and 3 trials such as SOLO3, OPINION, NORA, ARIEL4, and ATHENA [27, 60, 62, 82, 83], expanding the understanding of biomarker predictability for PARP inhibitor effectiveness. This updated review also covers biomarkers beyond the HRR pathway, such as ADP-Ribosylation [32], *HOXA9* promoter methylation [78], patient-derived organoids [73, 79], KELIM [33], and *SLFN11* [81].

The ESMO review featured 68 articles (66 primary research articles and 2 review articles), while this updated review included 103 articles (62 primary research articles and 41 review articles). Notably, only three research articles were included in both reviews due to the overlapping of certain clinical trials that were published in November and December 2019. The ESMO review primarily focused on early evidence concerning the predictive potential of biomarkers for platinum and PARP inhibitors. About one-third of the articles covered in the ESMO reviews centred on predictive biomarkers for platinum efficacy, with the remaining two-thirds concentrating on PARP inhibitors. Additionally, a third of the articles discussed other types of cancer. The ESMO review encompassed articles explaining concepts of testing assays (such as genomic scars and HRDetect scores). In contrast, this updated review places a stronger emphasis on primary research articles, predominantly concerning ovarian cancer and predictive biomarkers for PARP inhibitors, as detailed in Table 2 for a comparative summary.

**Fig. 3:**
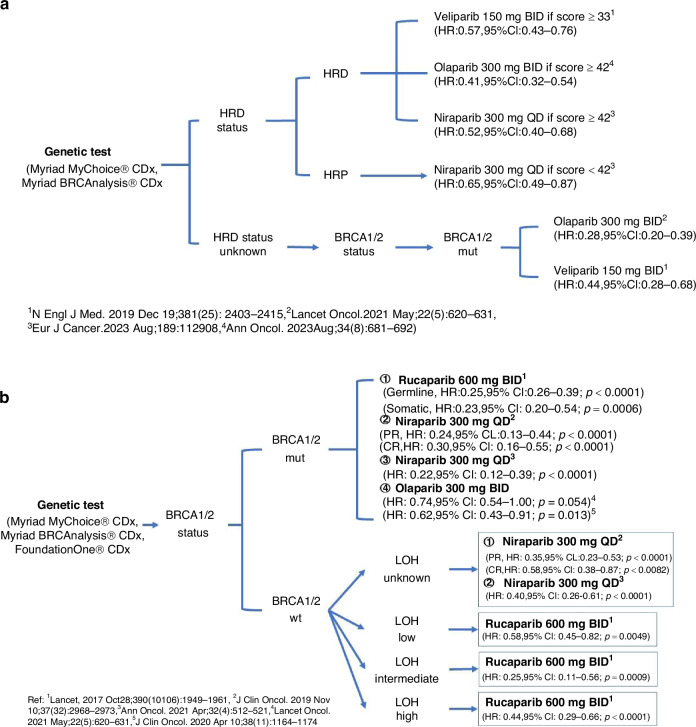
Decision-making framework for the use of PARP inhibitors in patients with ovarian cancer. **a** Decision-making framework for the use of PARP inhibitors in newly diagnosed advanced EOC. **b** Decision-making framework for the use of PARP inhibitors in platinum-sensitive recurrent EOC. mut mutation, BID twice daily, QD once daily.

Among the various biomarkers used to assess the effectiveness of PARP inhibitors in treating ovarian cancer, commercial genomic assays such as FoundationOne® CDx, and Myriad MyChoice® CDx are the primary ones employed in both clinical trials and clinical practice. Despite the binary classification system established around a threshold of 42, analysis has shown that patients with an HRD score ≥42 include a small proportion of patients with intact *BRCA1/2* genes, whereas those scoring <42 may also include some patients who exhibit *BRCA1/2* deficiencies. This finding helps explain why some ovarian cancer patients, even with an HRD score below 42, respond to PARP inhibitors [63]. Given the complexity of selecting PARP inhibitors for ovarian cancer patients, we propose a decision-making framework (Fig. 3) for newly diagnosed advanced EOC and platinum-sensitive EOC, based on evidence from Phase 3 randomised trials, to aid clinicians in understanding PARP inhibitor efficacy.

Current evidence underscores the clinical utility of HRR-related gene promoter methylation as a predictor of PARP inhibitor effectiveness [51, 52, 54, 55], yet larger prospective studies are vital for confirmation. Similarly, HRDetect, which utilises specific mutational signatures from cancer genome sequencing, shows promise in predictability [46, 54, 55] but requires further validation through extensive studies. The potential of real-time HRR pathway assessment, primarily through the RAD51 functional assay, in predicting PARP inhibitor efficacy is emerging from clinical research [46, 54, 55, 61, 73]; however, robust evidence from larger patient cohorts remains a prerequisite. While novel biomarkers outside the HRR pathway appear promising [32, 33, 73, 81], their validation in clinical studies is essential. Notably, the KELIM biomarker, already supported by post-hoc clinical trial analyses, necessitates prospective study validation to establish its utility.

The absence of a standardised approach for diagnosing HRD when comparing biomarkers remains a significant challenge. These biomarkers were originally developed to assist clinicians in stratifying patients for PARP inhibitor efficacy rather than precisely diagnosing HRD. Consequently, establishing a reference testing method to compare these predictive biomarkers’ performance effectively is complex. Rather than focusing solely on the detailed mechanisms of the HRR pathway, phenotypic outcomes following HRD, such as specific genomic scars and mutational signatures, show potential as standard diagnostic approaches. Additionally, functional studies, such as assessing RAD51 activity, can provide real-time information on the status of the HRR pathway. Together, these approaches may promise future incorporation as standard strategies for diagnosing HRD in the cancer genome.

In this systematic review, we adopted a similar approach to the original ESMO review (2020), conducting our literature search exclusively in the PubMed database. Expanding the search to additional medical databases, such as Embase and Scopus, could have provided a broader scope, potentially capturing further relevant evidence and ensuring a more comprehensive analysis. Compared to the ESMO review, which included 68 articles (66 primary research articles and 2 review articles), our updated review incorporates 103 articles, of which 62 are primary research articles and 41 are review articles. This increase in the number of review articles reflects an evolving and expanding body of literature on this topic, underscoring the ongoing research interest and the need for continuous updates in this field.

## Conclusion

Genomic assays for detecting *BRCA1/2* pathogenic variants and genomic scars are widely utilised as primary predictive biomarkers for assessing the efficacy of PARP inhibitors in ovarian cancer patients. While these assays provide valuable insights into HRD, the complexity of gene pathogenic variants and the multifaceted nature of genomic scars require further refinement in interpretation. Assessing the real-time functionality of HRR through nuclear RAD51 levels shows promise but necessitates additional investigation. Furthermore, novel biomarkers unrelated to HRD, such as ADP-ribosylation, *HOXA9* promoter methylation, patient-derived organoids, KELIM scores, and *SLFN11* expression, offer potential. Still, their clinical applicability and reliability require validation in large-scale studies. The quest for improved biomarkers to enhance PARP inhibitor response prediction in ovarian cancer patients continues, emphasising the need for standardised methodologies and comprehensive clinical validation to advance precision medicine in ovarian cancer treatment.

## Supplementary information

Appendix

## Data Availability

No datasets were generated or analysed during the current study.
