# Supplementary material for: Predictive biomarkers for the efficacy of PARP inhibitors in ovarian cancer: an updated systematic review
Source: BJC Rep. 2025 Mar 11;3:14. doi: 10.1038/s44276-025-00122-9 (PMC11897386; doi:10.1038/s44276-025-00122-9)
Supplement: Supplementary file 1 — Appendix [file 44276_2025_122_MOESM1_ESM.docx]

**Appendix**

**Predictive Biomarkers for the Efficacy of PARP Inhibitors in Ovarian Cancer: An Updated Systematic Review**

Ying-Wen Wang^1^, Isaac Allen^2^, Gabriel Funingana^3^, Marc Tischkowitz^4^, Yvonne Walburga^4^

^1^ Division of Gynaecologic Oncology, Department of Obstetrics and Gynaecology, Kaohsiung Chang Gung Memorial Hospital, Kaohsiung, Taiwan

^2^ Department of Public Health and Primary Care, University of Cambridge, Cambridge, England

^3^ Department of Oncology, University of Cambridge, Cambridge, England

^4^ Department of Medical Genetics, National Institute for Health Research Cambridge Biomedical Research Centre, University of Cambridge, Cambridge, UK.

Corresponding author: Ying-Wen Wang

Division of Gynaecologic Oncology, Department of Obstetrics and Gynaecology, Kaohsiung Chang Gung Memorial Hospital, Kaohsiung 833, Taiwan. E-mail: en7612@cgmh.org.tw

### Appendix Table 1: Summary of Search Strategies Used in the Systematic Review

| Description | Approach | last accessed | Search terms | ID | Result | Selection criteria | Number of articles retained after review |
| --- | --- | --- | --- | --- | --- | --- | --- |
| Screening literature to identify HRD tests employed alongside PARPi or platinum-based treatments | Pubmed search | 25.09.2019 - 31.03.2023 | (((HRD[Title/Abstract]) OR (BRCAness[Title/Abstract])) OR (homologous recombination[Title/Abstract])) AND (cancer[Title/Abstract]) AND (((test[Title/Abstract]) OR (biomarker[Title/Abstract])) OR (assay[Title/Abstract])) AND (((((((PARP inhibitor[Title/Abstract]) OR (cisplatin[Title/Abstract])) OR (carboplatin[Title/Abstract])) OR (oxaliplatin[Title/Abstract])) OR (olaparib[Title/Abstract])) OR (rucaparib[Title/Abstract])) OR (talazoparib[Title/Abstract])) AND 2019/09/25:2023/03/31[dp] | Main search | 127 | For evidence appraisal, the articles underwent a two-step screening process: all the article screen by titles and abstracts, followed by a full-text review for highly relevant articles; only original research (clinical and preclinical) pertaining to ovarian cancer and biomarkers associated with HRD retained | 13 |
| Screen all published trials of PARPi to identify the utilization of HRD tests | Pubmed search | 02.10.2019 - 31.03.2023 | olaparib AND 2019/10/02:2023/03/31[dp] (filter for clinical trial) | Olaparib | 106 | phase I studies and studies not directly relevant (e.g., pharmacodynamics or quality of life studies) were excluded; focus primarily on ovarian cancer, unless a study highly relevant for analytic/clinical validity assessment | 16 |
|  |  |  | rucaparib AND 2019/10/02:2023/03/31[dp] (filter for clinical trial) | Rucaparib | 27 |  | 8 |
|  |  |  | talazoparib AND 2019/10/02:2023/03/31[dp] (filter for clinical trial) | Talazoparib | 25 |  | 1 |
|  |  |  | niraparib AND 2019/10/02:2023/03/31[dp] (filter for clinical trial) | Niraparib | 31 |  | 6 |
|  |  |  | veliparib AND 2019/10/02:2023/03/31[dp] (filter for clinical trial) | Veliparib | 37 |  | 6 |
| Identify relevant review articles related to this topic | Pubmed search | 17.10.2019 - 31.03.2023 | (PARP inhibitors) AND (HRD) AND 2019/10/17:2023/03/31[dp] (filter for clinical trial and review) | Review | 77 | screen by titles and abstracts | 41 |
| Identify additional relevant original research through reviews | Screen citation lists of relevant reviews | NA | NA | Via_reviews | 8 | Likely to be relevant to evidence assessment based on title, abstract or text | 7 |
| Additional articles providing updated information on phase 3 studies | Search for updated information on phase 3 studies | NA | NA | Updated_Phase_3 | 5 |  | 5 |
|  |  |  |  | **Total records screened** | 443 | **Total articles included in the critical evidence review** | 103 |
|  |  |  |  |  |  | **Total original research articles included in the critical evidence review (excluding review articles)** | 62 |

### Appendix Table 2: Key Information of Articles Included in this Systematic Review

| Search category | Title | Study ID (Clinical trial registration no) | Acronym | Reference | Study type | Study cohorts or Participants | Setting or Clinical scenario | Drug | Details regarding HRD test | Category of HRD test | LOE category | Analytic validity (EGAPP) | Clinical validity (EGAPP) | Clinical Utility (EGAPP) |
| --- | --- | --- | --- | --- | --- | --- | --- | --- | --- | --- | --- | --- | --- | --- |
| Veliparib | Veliparib with First-Line Chemotherapy and as Maintenance Therapy in Ovarian Cancer | NCT02470585 | VELIA | N Engl J Med. 2019 Dec 19;381(25): 2403-2415 | Phase III RCT | Newly diagnosed stage III or IV, HGSC | Frontline maintenance, 1:1:1 (control: veliparib combination: veliparib throughout) | Veliparib | Genetic test done after enrolment; germline or somatic BRCA1/2 pathogenic variant determined by Myriad BRACAnalysis CDx or myChoice HRD CDx assay; HRD positive defined as BRCA mutant or myChoice HRD CDx assay; positive when score at least 33) | 1,2 | A | 1 | 1 | 2 |
| Niraparib | Niraparib Maintenance Therapy in Patients With Recurrent Ovarian Cancer After a Partial Response to the Last Platinum-Based Chemotherapy in the ENGOT-OV16/NOVA Trial | NCT01847274 | NOVA | J Clin Oncol. 2019 Nov 10;37(32):2968-2973. | Phase III RCT | Platinum-sensitive recurrent | Salvage maintenance, 2:1 (niraparib: control) | Niraparib | Genetic test done after enrolment; germline BRCA1/2 pathogenic variant determined by Myriad BRACAnalysis CDx | 1 | A | 1 | 1 | 2 |
| Niraparib | Niraparib in Patients with Newly Diagnosed Advanced Ovarian Cancer | NCT02655016 | PRIMA | N Engl J Med. 2019 Dec 19;381(25):2391-2402. | Phase III RCT | Newly diagnosed stage III or IV, high-grade serous or endometrioid type | Frontline maintenance, 2:1 (niraparib: control) | Niraparib | Genetic test done after enrolment; HRD status determined by the Myriad myChoice HRD CDx assay; HRD positive when score at least 42) | 1,2 | A | 1 | 1 | 2 |
| Rucaparib | Antitumor activity of the poly(ADP-ribose) polymerase inhibitor rucaparib as monotherapy in patients with platinum-sensitive, relapsed, BRCA-mutated, high-grade ovarian cancer, and an update on safety | NCT01482715, NCT01891344 | Study 10 and ARIEL2 | Int J Gynecol Cancer. 2019 Nov;29(9):1396-1404. | Integrated analysis of 2 single-arm phase II studies | Platinum-sensitive recurrent with high-grade serous or endometrioid type | Salvage | Rucaparib | All enrolled patients in this integrated analysis had known *BRCA1/2* mutation; In study 10: all enrolled patients had known germline BRCA mutation, determined by a local laboratory; In ARIEL2: all enrolled patients with tumour tested by Foundation Medicine T5 next-generation sequencing assay (for BRCA1/2 and LOH; cut-off for LOH is 14%); methylation-sensitive PCR done for the promoter methylation of RAD51C and BRCA1; germline or somatic alterations also determined by BROCA-homologous recombination sequencing assay | 1,2 | B | 1 | 1 | 3 |
| Olaparib | Olaparib maintenance monotherapy in platinum-sensitive, relapsed ovarian cancer without germline BRCA mutations: OPINION Phase IIIb study design | NCT03402841 | OPINION | Future Oncol. 2019 Nov;15(32):3651-3663. | Phase IIIb, single-arm | Platinum-sensitive recurrent with HGSC or endometrioid type without germline BRCA mutation | Salvage | Olaparib | All enrolled patients did not have germline BRCA mutation, determined by the Myriad BRCAnalysis CDx; further somatic BRCA and HRD status be determined by the Myriad myChoice HRD plus test, HRD positive when score at least 42) | 1,2 | NA | NA | NA | NA |
| Olaparib | Olaparib plus Bevacizumab as First-Line Maintenance in Ovarian Cancer | NCT02477644 | PAOLA-1 | N Engl J Med. 2019 Dec 19;381(25):2416-2428. | Phase III RCT | Newly diagnosed stage III or IV, high-grade serous or endometrioid type | Frontline maintenance, 2:1 (olaparib: control) | Olaparib | Tumour BRCA and HRD status determined by the Myriad myChoice HRD Plus assay (HRD positive if BRCA mutation or score at least 42) | 1,2 | A | 1 | 1 | 2 |
| Main search | ADP-Ribosylation Levels and Patterns Correlate with Gene Expression and Clinical Outcomes in Ovarian Cancers |  |  | Mol Cancer Ther. 2020 Jan;19(1):282-291. | Retrospective | 34 EOC patients with HGSC | Cancer cells derived from enrolled patients, examining the ADP-Ribosylation Levels and response to olaparib, then linking the results with clinical outcome | Olaparib | Tumour sample analysed for ADP-Ribosylation Levels, then linking to patients' clinical data and sensitivity of PARPi | 4 | D | 2 | 2 | 3 |
| Niraparib | Immunogenomic profiling determines responses to combined PARP and PD-1 inhibition in ovarian cancer | NCT02657889 | TOPACIO | Nat Commun. 2020 Mar 19;11(1):1459. | Phase I/II trial (archived samples used for testing) | 62 Recurrent EOC, mostly platinum-resistant | Salvage, Single-arm | Niraparib + pembrolizumab | Myriad myChoice HRD assay used to determine BRCA and HRD status; BROCA test also used to determine 84 DNA repair genes and the methylation status BRCA1 and RAD51C; mutational signature also determined; IHC of RAD51; FISH of PD-L1 and PD-L2 | 1,2,3 | B | 1 | 1 | 3 |
| Olaparib | Olaparib Versus Nonplatinum Chemotherapy in Patients With Platinum-Sensitive Relapsed Ovarian Cancer and a Germline BRCA1/2 Mutation (SOLO3): A Randomized Phase III Trial | NCT02282020 | SOLO3 | J Clin Oncol. 2020 Apr 10;38(11):1164-1174 | Phase III RCT | Platinum-sensitive recurrent, high-grade serous or endometrioid | Salvage, 2:1 (olaparib: single nonplatinum chemotherapy) | Olaparib | Patients all had known germline BRCA mutation by local lab and confirmed by the Myriad BRCAnalysis CDx | 1 | A | 1 | 1 | 2 |
| Veliparib | Circulating HOXA9-methylated tumour DNA: A novel biomarker of response to poly (ADP-ribose) polymerase inhibition in BRCA-mutated epithelial ovarian cancer | NCT01472783 |  | Eur J Cancer. 2020 Jan;125:121-129 | Phase II | 32 Platinum-resistant recurrent with known BRCA-mutated | Salvage | Veliparib | Patients all had BRCA mutation (not clearly mention how the BRCA status determined); HOXA9 promoter methylation status determined by HOXA9 methylation-specific assay | 1,4 | B | 1 | 1 | 3 |
| Main search | BRCA Methylation Testing Identifies a Subset of Ovarian Carcinomas without Germline Variants That Can Benefit from PARP Inhibitor |  |  | Int J Mol Sci. 2020 Dec 19;21(24):9708 | Retrospective (linking methylation status to outcome) | 90 EOC without germline BRCA mutation |  |  | All patients do not have germline BRCA mutation, tumour BRCA1/2 promoter methylation status determined by EZ DNA Methylation kit and pyrosequencing | 1 | D | 2 | 2 | 3 |
| Main search | Development of a 3D functional assay and identification of biomarkers, predictive for response of high-grade serous ovarian cancer (HGSOC) patients to poly-ADP ribose polymerase inhibitors (PARPis): targeted therapy |  |  | J Transl Med. 2020 Nov 19;18(1):439 | Preclinical study | 83 patients with EOC, ascites-derived primary cell cultures (AsPC) |  | Niraparib or Olaparib | 3D organoid developed from ascites cell culture; microarray test done in these organoids and selected 7 potential biomarkers for predicting the sensitivity to PARPi | 4 | ? (Preclinical study) | 2 | 2 | 3 |
| Olaparib | Maintenance olaparib for patients with newly diagnosed advanced ovarian cancer and a BRCA mutation (SOLO1/GOG 3004): 5-year follow-up of a randomised, double-blind, placebo-controlled, phase 3 trial | NCT01844986 | SOLO1 | Lancet Oncol. 2021 Dec;22(12):1721-1731. | Phase III RCT | Newly diagnosed stage III or IV, high-grade serous or endometrioid | Frontline maintenance, 2:1 (Olaparib:Placebo) | Olaparib | All patients having known germline or tumour BRCA mutation (test by local lab, Myriad BRACAnalysis CDx or the BRCA1 and BRCA2 genetic testing assay (Beijing Genomics Institute, China). | 1 | A | 1 | 1 | 2 |
| Rucaparib | Rucaparib maintenance treatment for recurrent ovarian carcinoma: the effects of progression-free interval and prior therapies on efficacy and safety in the randomized phase III trial ARIEL3 | NCT01968213 | ARIEL3 | Int J Gynecol Cancer. 2021 Jul;31(7):949-958 | Phase III RCT | Platinum-sensitive recurrent, high-grade serous or endometrioid | Salvage maintenance, 2:1 (rucaparib: control) | Rucaparib | Genetic tests done after enrolment; all enrolled patients with tumour tested by Foundation Medicine T5 next-generation sequencing assay (for BRCA1/2 and LOH; cut-off for LOH is 16%); and Germline BRCA mutations identified by Myriad BRCAnalysis CDx | 1,2 | A | 1 | 1 | 2 |
| Olaparib | Olaparib plus bevacizumab as maintenance therapy in patients with newly diagnosed, advanced ovarian cancer: Japan subset from the PAOLA-1/ENGOT-ov25 trial | NCT02477644 | PAOLA-1 | J Gynecol Oncol. 2021 Sep;32(5):e82 | Phase III RCT | Newly diagnosed stage III or IV, high-grade serous or endometrioid type | Frontline maintenance, 2:1 (olaparib: control) | Olaparib | Tumour BRCA and HRD status determined by the Myriad myChoice HRD Plus assay (HRD positive if BRCA mutation or score at least 42) | 1,2 | A | 1 | 1 | 2 |
| Rucaparib | Maintenance treatment with rucaparib for recurrent ovarian carcinoma in ARIEL3, a randomized phase 3 trial: The effects of best response to last platinum-based regimen and disease at baseline on efficacy and safety | NCT01968213 | ARIEL3 | Cancer Med. 2021 Oct;10(20):7162-7173 | Phase III RCT | Platinum-sensitive recurrent, high-grade serous or endometrioid | Salvage maintenance, 2:1 (rucaparib: control) | Rucaparib | Genetic tests done after enrolment; all enrolled patients with tumour tested by Foundation Medicine T5 next-generation sequencing assay (for BRCA1/2 and LOH; cut-off for LOH is 16%); and Germline BRCA mutations identified by Myriad BRCAnalysis CDx | 1,2 | A | 1 | 1 | 2 |
| Niraparib | Phase 2 single-arm study on the efficacy and safety of niraparib in Japanese patients with heavily pretreated, homologous recombination-deficient ovarian cancer | NCT03759600 | QUADRA | J Gynecol Oncol. 2021 Mar;32(2):e16 | Phase II, single-arm | 20 Platinum-sensitive recurrent, HGSC, HRD positive | Salvage | Niraparib | Tumour evaluated by Myriad myChoice HRD CDx (HRD positive if BRCA mutation or score at least 42) | 1,2 | B | 1 | 1 | 3 |
| Olaparib | Olaparib tablets as maintenance therapy in patients with platinum-sensitive relapsed ovarian cancer and a BRCA1/2 mutation (SOLO2/ENGOT-Ov21): a final analysis of a double-blind, randomised, placebo-controlled, phase 3 trial | NCT01874353 | SOLO2 | Lancet Oncol. 2021 May;22(5):620-631 | Phase III RCT | Platinum-sensitive recurrent, all with germline or somatic BRCA mutation | Salvage maintenance, 2:1 (Olaparib:Placebo) | Olaparib | All patients have known germline or tumour BRCA mutation, confirmed by the Myriad BRCAnalysis CDx | 1 | A | 1 | 1 | 2 |
| Rucaparib | Molecular and clinical determinants of response and resistance to rucaparib for recurrent ovarian cancer treatment in ARIEL2 (Parts 1 and 2) | NCT01891344 | ARIEL2 | Nat Commun. 2021 May 3;12(1):2487 | Phase II, single-arm | Platinum-sensitive recurrent with HGSC or endometrioid type | Salvage | Rucaparib | Genetic tests done after enrolment; all enrolled patients with tumour tested by Foundation Medicine T5 next-generation sequencing assay (for BRCA1/2 and LOH; cut-off for LOH is 14%); methylation-sensitive PCR also done for the promoter methylation of RAD51C and BRCA1; germline alterations also determined by BROCA-homologous recombination sequencing assay | 1,2 | B | 1 | 1 | 3 |
| Olaparib | Olaparib maintenance therapy in patients with newly diagnosed advanced ovarian cancer and a BRCA1 and/or BRCA2 mutation: SOLO1 China cohort | NCT01844986 | SOLO1 | Gynecol Oncol. 2021 Jan;160(1):175-181 | Phase III RCT | Newly diagnosed stage III or IV, high-grade serous or endometrioid | Frontline maintenance, 2:1 (Olaparib:Placebo) | Olaparib | All patients having known germline or tumour BRCA mutation (test by local lab, Myriad BRACAnalysis CDx or the BRCA1 and BRCA2 genetic testing assay (Beijing Genomics Institute, China). | 1 | A | 1 | 1 | 2 |
| Niraparib | Niraparib maintenance therapy in patients with platinum-sensitive recurrent ovarian cancer using an individualized starting dose (NORA): a randomized, double-blind, placebo-controlled phase III trial | NCT03705156 | NORA | Ann Oncol. 2021 Apr;32(4):512-521. | Phase III RCT | Platinum-sensitive recurrent, mainly HGSC | Salvage maintenance, 2:1 (niraparib: control) | Niraparib | Germline BRCA status will be determined after enrolment | 1 | A | 1 | 1 | 2 |
| Veliparib | Impact of veliparib, paclitaxel dosing regimen, and germline BRCA status on the primary treatment of serous ovarian cancer - an ancillary data analysis of the VELIA trial | NCT02470585 | VELIA | Gynecol Oncol. 2022 Feb;164(2):278-287 | Phase III RCT | Newly diagnosed stage III or IV, HGSC | Frontline maintenance, 1:1:1 (control: veliparib combination: veliparib throughout) | Veliparib | Genetic test done after enrolment; germline or somatic BRCA1/2 pathogenic variant determined by Myriad BRACAnalysis CDx or myChoice HRD CDx assay; HRD positive defined as BRCA mutant or myChoice HRD CDx assay; positive when score at least 33) | 1,2 | A | 1 | 1 | 2 |
| Olaparib | Olaparib treatment for platinum-sensitive relapsed ovarian cancer by BRCA mutation and homologous recombination deficiency status: Phase II LIGHT study primary analysis | NCT02983799 | LIGHT | Gynecol Oncol. 2022 Sep;166(3):425-431 | Phase II, single-arm | Platinum-sensitive recurrent, high-grade serous or endometrioid | Salvage | Olaparib | Tumours analysed by Myriad BRACAnalysis CDx and myChoice HRD assays; HRD positive BRCA1/2 mutation or genomic instability score at least 42 | 1,2 | B | 1 | 1 | 3 |
| Main search | Alternative academic approaches for testing homologous recombination deficiency in ovarian cancer in the MITO16A/MaNGO-OV2 trial | NCT01706120 | MITO16A | ESMO Open. 2022 Oct;7(5):100585 | Phase IV | 100 newly diagnosed EOC, HGSC | Frontline | Carboplatin+Paclitaxel+Bev | Tumour analysis by LAB1, LAB2 or LAB3 and compared to Myriad myChoice HRD assay (LAB1: Agilent OneSeq Constitutional Panel, HRD score at least 42; LAB2: Illumina TS170; LAB3: Immunofluorescence of RAD51, HRD if RAD51 score less than 10%) | 1,2,3 | B | 1 | 1 | 3 |
| Main search | Overlapping gene dependencies for PARP inhibitors and carboplatin response identified by functional CRISPR-Cas9 screening in ovarian cancer |  |  | Cell Death Dis. 2022 Oct 28;13(10):909. | Preclinical study | EOC cell line |  | Olaparib, Niraparib, Talazoparib, Carboplatin | CRISPR-Cas9 used in EOC cell lines and evaluating 93 candidate genes and their predictability to the response of PARPi | 1 | ? (Preclinical study) | 3 | 4 | 3 |
| Olaparib | Efficacy of subsequent chemotherapy for patients with BRCA1/2-mutated recurrent epithelial ovarian cancer progressing on olaparib versus placebo maintenance: post-hoc analyses of the SOLO2/ENGOT Ov-21 trial | NCT01874353 | SOLO2 | Ann Oncol. 2022 Oct;33(10):1021-1028 | Phase III RCT | Platinum-sensitive recurrent, all with germline or somatic BRCA mutation | Salvage maintenance, 2:1 (Olaparib:Placebo) | Olaparib | All patients have known germline or tumour BRCA mutation, confirmed by the Myriad BRCAnalysis CDx | 1 | A | 1 | 1 | 2 |
| Main search | Basal expression of RAD51 foci predicts olaparib response in patient-derived ovarian cancer xenografts |  |  | Br J Cancer. 2022 Jan;126(1):120-128 | Preclinical study | 47 ovarian cancer patient-derived-xenograft |  | Olaparib + Cisplatin | All PDX evaluated for BRCA1/2 mutation and HRDetect score by whole genome sequencing, BRCA1 promoter methylation; IHC for RAD51 and γH2AX, mRNA expression of 21 selected genes, CCNE1 gene copy number, and immunofluorescence for RAD51 and γH2AX | 1,2,3,4 | ? (Preclinical study) | 2 | 2 | 3 |
| Niraparib | OVARIO phase II trial of combination niraparib plus bevacizumab maintenance therapy in advanced ovarian cancer following first-line platinum-based chemotherapy with bevacizumab | NCT03326193 | OVARIO | Gynecol Oncol. 2022 Aug;166(2):219-229 | Phase II, single-arm | Newly diagnosed stage III-IV, high-grade serous or endometrioid | Frontline maintenance after response to platinum-based chemotherapy | Niraparib + Bev | Tumour BRCA and HRD status as determined by the Myriad myChoice HRD test (HRD positive when score at least 42) | 1,2 | B | 1 | 1 | 3 |
| Olaparib | Efficacy of maintenance olaparib plus bevacizumab according to clinical risk in patients with newly diagnosed, advanced ovarian cancer in the phase III PAOLA-1/ENGOT-ov25 trial | NCT02477644 | PAOLA-1 | Gynecol Oncol. 2022 Feb;164(2):254-264 | Phase III RCT | Newly diagnosed stage III or IV, high-grade serous or endometrioid type | Frontline maintenance, 2:1 (olaparib: control) | Olaparib | Tumour BRCA and HRD status determined by the Myriad myChoice HRD Plus assay (HRD positive if BRCA mutation or score at least 42) | 1,2 | A | 1 | 1 | 2 |
| Main search | Prediction of homologous recombination deficiency from cancer gene expression data |  |  | J Int Med Res. 2022 Nov;50(11):3000605221133655 | Data analysis, derived from TCGA |  |  |  | Three components of the HRD score are from gene expression: 1. large (>15Mb) non arm-level regions with LOH, 2. large-scale state transitions (breaks between adjacent segments of >10Mb), and 3. Sub-telomeric regions with allelic imbalance. | 2 | B (?) | 1 | 1 | ? |
| Rucaparib | Rucaparib versus standard-of-care chemotherapy in patients with relapsed ovarian cancer and a deleterious BRCA1 or BRCA2 mutation (ARIEL4): an international, open-label, randomised, phase 3 trial | NCT02855944 | ARIEL4 | Lancet Oncol. 2022 Apr;23(4):465-478. | Phase III RCT | BRCA-mutated recurrent ovarian cancer (including platinum-sensitive and platinum-resistant) | Salvage, 2:1 (Rucaparib:Chemotherapy) | Rucaparib | All enrolled patients had known germline or somatic BRCA1/2 mutation: FoundationOne NGS assay to detect tumour BRCA mutations, BRCA reversion mutations prospectively tested in cell-free DNA collected before rucaparib by Guardant Health’s Guardant360 NGS assay, Central germline BRCA testing done with Ambry Genetics’ CancerNext test (Aliso Viejo, CA, USA) | 1 | A | 1 | 1 | 2 |
| Olaparib | Biomarker-guided targeted therapy in platinum-resistant ovarian cancer (AMBITION; KGOG 3045): a multicentre, open-label, five-arm, uncontrolled, umbrella trial | NCT03699449 | AMBITION | J Gynecol Oncol. 2022 Jul;33(4):e45. | Phase II, umbrella trial | Platinum-resistant recurrent | Patient assigned an tx based on the status of biomarkers (HRD, PD-L1) | Olaparib + Cediranib or Olaparib + Durvalumab | PD-L1 and HRD were examined in tumour samples; HRD determined by a HRR-gene panel (including 15 HRR-related genes) | 1 | B | 1 | 1 | 3 |
| Olaparib | Olaparib maintenance monotherapy in Chinese patients with platinum-sensitive relapsed ovarian cancer: China cohort from the phase III SOLO2 trial | NCT01874353 | SOLO2 | Asia Pac J Clin Oncol. 2022 Dec;18(6):714-722. | Phase III RCT | Platinum-sensitive recurrent, all with germline or somatic BRCA mutation | Salvage maintenance, 2:1 (Olaparib:Placebo) | Olaparib | All patients have known germline or tumour BRCA mutation, confirmed by the Myriad BRCAnalysis CDx | 1 | A | 1 | 1 | 2 |
| Olaparib | Olaparib With or Without Cediranib Versus Platinum-Based Chemotherapy in Recurrent Platinum-Sensitive Ovarian Cancer (NRG-GY004): A Randomized, Open-Label, Phase III Trial | NCT02446600 | NRG-GY004 | J Clin Oncol. 2022 Jul 1;40(19):2138-2147. | Phase III RCT | Platinum sensitive recurrent, high-grade serous or endometrioid | Salvage, 1:1:1 (platinum-based chemotherap: olaparib: olaparib/cediranib | Olaparib with or without cediranib | Germline BRCA status will be determined after enrolment (Myriad BRCAnalysis CDx) | 1 | A | 1 | 1 | 2 |
| Rucaparib | A Randomized, Phase III Trial to Evaluate Rucaparib Monotherapy as Maintenance Treatment in Patients With Newly Diagnosed Ovarian Cancer (ATHENA-MONO/GOG-3020/ENGOT-ov45) | NCT03522246 | ATHENA-MONO | J Clin Oncol. 2022 Dec 1;40(34):3952-3964. | Phase III RCT | Newli diagnosed stage III-IV, high-grade | Frontline maintenance, 4:1 (rucaparib:placebo) | Rucaparib | Tumour HRD (BRCA mutations and genomic LOH) was determined by the FoundationOne CDx assay | 1,2 | A | 1 | 1 | 2 |
| Rucaparib | Clinical and molecular characteristics of ARIEL3 patients who derived exceptional benefit from rucaparib maintenance treatment for high-grade ovarian carcinoma | NCT01968213 | ARIEL3 | Gynecol Oncol. 2022 Dec;167(3):404-413 | Phase III RCT | Platinum-sensitive recurrent, high-grade serous or endometrioid | Salvage maintenance, 2:1 (rucaparib: control) | Rucaparib | Genetic tests done after enrolment; all enrolled patients with tumour tested by Foundation Medicine T5 next-generation sequencing assay (for BRCA1/2 and LOH; cut-off for LOH is 16%); and Germline BRCA mutations identified by Myriad BRCAnalysis CDx | 1,2 | A | 1 | 1 | 2 |
| Main search | Preclinical In Vivo Validation of the RAD51 Test for Identification of Homologous Recombination-Deficient Tumors and Patient Stratification |  |  | Cancer Res. 2022 Apr 15;82(8):1646-1657. | Preclinical study | Patient-derived xenograft (4 Ovarian cancer, HGSC) |  | Olaparib | Cells examined for genomic scar (by Myriad myChoice HRD CDx or HRDetect), BRCA1 promoter methylation by MS-MLPA, BRCA1 mRNA expression, and Immunofluorescence for RAD51 | 1,2,3 | ? (Preclinical study) | 1 | 2 | 3 |
| Olaparib | Olaparib maintenance monotherapy in platinum-sensitive relapsed ovarian cancer patients without a germline BRCA1/BRCA2 mutation: OPINION primary analysis | NCT03402841 | OPINION | Gynecol Oncol. 2022 Mar;164(3):498-504. | Phase IIIb, single-arm | Platinum-sensitive recurrent with high-grade serous or endometrioid without germline BRCA mutation | Salvage | Olaparib | All enrolled patients did not have germline BRCA mutation, determined by the Myriad BRCAnalysis CDx; further somatic BRCA and HRD status be determined by the Myriad myChoice HRD plus test, HRD positive when score at least 42) | 1,2 | A | 1 | 1 | 2 |
| Veliparib | Impact of homologous recombination status and responses with veliparib combined with first-line chemotherapy in ovarian cancer in the Phase 3 VELIA/GOG-3005 study | NCT02470585 | VELIA | Gynecol Oncol. 2022 Feb;164(2):245-253 | Phase III RCT | Newly diagnosed stage III or IV, HGSC | Frontline maintenance, 1:1:1 (control: veliparib combination: veliparib throughout) | Veliparib | Genetic test done after enrolment; germline or somatic BRCA1/2 pathogenic variant determined by Myriad BRACAnalysis CDx or myChoice HRD CDx assay; HRD positive defined as BRCA mutant or myChoice HRD CDx assay; positive when score at least 33) | 1,2 | A | 1 | 1 | 2 |
| Main search | Developing patient-derived organoids to predict PARP inhibitor response and explore resistance overcoming strategies in ovarian cancer |  |  | Pharmacol Res. 2022 May;179:106232. | Case series | 7 patient-derived organoids of primary or recurrent EOC |  | Olaparib, Niraparib, Cisplatin | All samples (organoids) analysed by whole exome sequencing; Immunofluorescence for DAPI, germinin, RAD51 and histone H2AX | 3,4 | C (?, preclinical study) | 3 | 4 | 4 |
| Main search | Homologous recombination deficiency in diverse cancer types and its correlation with platinum chemotherapy efficiency in ovarian cancer |  |  | BMC Cancer. 2022 May 16;22(1):550. | Prospective and retrospective | 85 patients with EOC (75.3% platinum-sensitive, BRCA-mutant 32.9%) |  | Platinum | All tumour samples evaluated by a HRD gene panel (GeneseeqPrime HRD panel targeting 425 cancer-relevant genes), HRD positive when score at least 38 (based on LOH, TAI, LST) | 2 | B | 1 | 1 | 3 |
| Main search | Mathematical modelling of the early modelled CA-125 longitudinal kinetics (KELIM-PARP) as a pragmatic indicator of rucaparib efficacy in patients with recurrent ovarian carcinoma in ARIEL2 & STUDY 10 | NCT01482715, NCT01891344 | Study 10 and ARIEL2 | EBioMedicine. 2023 Mar;89:104477. | Retrospective studies of 2 phase II studies | Platinum-sensitive or platinum-resistant recurrent with high-grade serous or endometrioid | Salvage | Rucaparib | HRD status determined by BRCA1/2 mutation and/or genomic LOH at least 16% by the Foundation Medicine next-generation sequencing assay; also evaluating the predictability of KELIM-PARP | 1,2,4 | B | 1 | 1 | 3 |
| Olaparib | Overall Survival With Maintenance Olaparib at a 7-Year Follow-Up in Patients With Newly Diagnosed Advanced Ovarian Cancer and a BRCA Mutation: The SOLO1/GOG 3004 Trial | NCT01844986 | SOLO1 | J Clin Oncol. 2023 Jan 20;41(3):609-617 | Phase III RCT | Newly diagnosed stage III or IV, high-grade serous or endometrioid | Frontline maintenance, 2:1 (Olaparib:Placebo) | Olaparib | All patients having known germline or tumour BRCA mutation (test by local lab, Myriad BRACAnalysis CDx or the BRCA1 and BRCA2 genetic testing assay (Beijing Genomics Institute, China). | 1 | A | 1 | 1 | 2 |
| Rucaparib | Efficacy and safety of rucaparib treatment in patients with BRCA-mutated, relapsed ovarian cancer: final results from Study 10 | NCT01482715 | Study 10 | Br J Cancer. 2023 Jan;128(2):255-265. | Phase II, single-arm | Recurrent with high-grade serous or endometrioid | Salvage | Rucaparib | All enrolled patients had known germline BRCA mutation, determined by a local laboratory | 1 | B | 1 | 1 | 3 |
| Veliparib | Veliparib with frontline chemotherapy and as maintenance in Japanese women with ovarian cancer: a subanalysis of efficacy, safety, and antiemetic use in the phase 3 VELIA trial | NCT02470585 | VELIA | Int J Clin Oncol. 2023 Jan;28(1):163-174 | Phase III RCT | Newly diagnosed stage III or IV, HGSC | Frontline maintenance, 1:1:1 (control: veliparib combination: veliparib throughout) | Veliparib | Genetic test done after enrolment; germline or somatic BRCA1/2 pathogenic variant determined by Myriad BRACAnalysis CDx or myChoice HRD CDx assay; HRD positive defined as BRCA mutant or myChoice HRD CDx assay; positive when score at least 33) | 1,2 | A | 1 | 1 | 2 |
| Olaparib | Homologous Recombination Repair Gene Mutations to Predict Olaparib Plus Bevacizumab Efficacy in the First-Line Ovarian Cancer PAOLA-1/ENGOT-ov25 Trial | NCT02477644 | PAOLA-1 | JCO Precis Oncol. 2023 Jan;7:e2200258. | Phase III RCT | Newly diagnosed stage III or IV, high-grade serous or endometrioid type | Frontline maintenance, 2:1 (olaparib: control) | Olaparib | Tumour BRCA and HRD status determined by the Myriad myChoice HRD Plus assay (HRD positive if BRCA mutation or score at least 42); archival samples further evaluated by a non-BRCA HRR gene panels | 1,2 | A | 1 | 1 | 2 |
| Veliparib | Molecular Profiling-Based Assignment of Cancer Therapy (NCI-MPACT): A Randomized Multicenter Phase II Trial | NCT01827384 | NCI-MPACT | JCO Precis Oncol. 2021 Jan 12;5:PO.20.00372 | Phase II | 96 patients with solid tumours | Salvage | Everolimus, trametinib, veliparib + temozolomide, or adavosertib + carboplatin (only 5 patients receiving veliparib + temozolomide) | Enrolled patients all having actionable mutation in DNA repair, RAS/RAF/MEK, or AKT/PI3K/MTOR pathways (examined by a targeted gene panel, with a mutation frequency of 5%) | 4 | A (?) | 2 | 2 | 2 |
| Main search | hMOB2 deficiency impairs homologous recombination-mediated DNA repair and sensitises cancer cells to PARP inhibitors |  |  | Cell Signal. 2021 Nov;87:110106 | Preclinical study | Ovarian cancer cell line |  | Olaparib, veliparib, rucaparib | Examining the hMOB2 function by knockout; alkaline comet assay to determine the quantity of double-strand break; clonogenic survival assay evaluating cell survival; GFP-based DNA repair reporter assays determine the efficacy of HR and NHEJ | 1 | ? (Preclinical study) | 2 | 2 | 3 |
| Main search | The disruption of the CCDC6 - PP4 axis induces a BRCAness like phenotype and sensitivity to PARP inhibitors in high-grade serous ovarian carcinoma |  |  | J Exp Clin Cancer Res. 2022 Aug 13;41(1):245 | Preclinical study | Cells of HGSC |  | Olaparib | Developing olaparib-resistant cell line, and make cell lines with CCDC6-depletion; cell lines can regain the sensitivity to PARPi after CCDC6-depletion | 1 | ? (Preclinical study) | 2 | 2 | 3 |
| Talazoparib | PARP Inhibitor in Platinum-Resistant Ovarian Cancer: Single-Center Real-World Experience |  |  | JCO Glob Oncol. 2021 Apr;7:506-511 | Retrospective, single-arm | 28 recurrent HGSC (including platinum-resistant and platinum sensitive); known germline BRCA mutation | Salvage | Olaparib or Talazoparib | All patients have germline BRCA mutation (testing method not mentioned in the article) | 1 | D | 2 | 2 | 3 |
| Updated_Phase_3 | Olaparib plus bevacizumab first-line maintenance in ovarian cancer: final overall survival results from the PAOLA-1/ENGOT-ov25 trial | NCT02477644 | PAOLA-1 | Ann Oncol. 2023 Aug;34(8):681-692. | Phase III RCT | Newly diagnosed stage III or IV, high-grade serous or endometrioid type | Frontline maintenance, 2:1 (olaparib: control) | Olaparib | Tumour BRCA and HRD status determined by the Myriad myChoice HRD Plus assay (HRD positive if BRCA mutation or score at least 42) | 1,2 | A | 1 | 1 | 2 |
| Updated_Phase_3 | PARP inhibitor predictive value of the Leuven HRD test compared with Myriad MyChoice CDx PLUS HRD on 468 ovarian cancer patients from the PAOLA-1/ENGOT-ov25 trial | NCT02477644 | PAOLA-1 | Eur J Cancer. 2023 Jul;188:131-139. | Phase III RCT | Newly diagnosed stage III or IV, high-grade serous or endometrioid type | Frontline maintenance, 2:1 (olaparib: control) | Olaparib | Tumour BRCA and HRD status determined by the Myriad myChoice HRD Plus assay (HRD positive if BRCA1/2 mutation or score at least 42) and Leuven HRD assay (HRD posotive if BRCA1/2 mutation or GIS at least 56) | 1,2 | A | 1 | 1 | 2 |
| Updated_Phase_3 | Normalized LST Is an Efficient Biomarker for Homologous Recombination Deficiency and Olaparib Response in Ovarian Carcinoma | NCT02477644 | PAOLA-1 | JCO Precis Oncol. 2023 Jun;7:e2200555. | Phase III RCT | Newly diagnosed stage III or IV, high-grade serous or endometrioid type | Frontline maintenance, 2:1 (olaparib: control) | Olaparib | Tumour BRCA and HRD status determined by the Myriad myChoice HRD Plus assay (HRD positive if BRCA mutation or score at least 42) and Geneva test (OncoScan+nLST) | 1,2 | A | 1 | 1 | 2 |
| Updated_Phase_3 | Concordance Between Tumour and Germline BRCA Status in High-Grade Ovarian Carcinoma Patients in the Phase III PAOLA-1/ENGOT-ov25 Trial | NCT02477644 | PAOLA-1 | J Natl Cancer Inst. 2021 Jul 1;113(7):917-923 | Phase III RCT | Stage III or IV, high-grade serous or endometrioid type | Frontline maintenance, 2:1 (olaparib: control) | Olaparib | Tumour BRCA and HRD status determined in the original PAOLA-1 trial; HRD defined as (score at least 42 on the myChoice HRD Plus assay or tumour BRCA mutation) | 1,2 | A | 1 | 1 | 2 |
| Updated_Phase_3 | Progression-free survival and safety at 3.5years of follow-up: results from the randomised phase 3 PRIMA/ENGOT-OV26/GOG-3012 trial of niraparib maintenance treatment in patients with newly diagnosed ovarian cancer | NCT02655016 | PRIMA | Eur J Cancer. 2023 Aug;189:112908. | Phase III RCT | Newly diagnosed stage III or IV, high-grade serous or endometrioid type | Frontline maintenance, 2:1 (niraparib: control) | Niraparib | Genetic test done after enrolment; HRD status determined by the Myriad myChoice HRD CDx assay; HRD positive when score at least 42) | 1,2 | A | 1 | 1 | 2 |
| Via_reviews | Genomic profiling in ovarian cancer retreated with platinum based chemotherapy presented homologous recombination deficiency and copy number imbalances of CCNE1 and RB1 genes |  |  | BMC Cancer. 2019 May 6;19(1):422 | Retrospective | 31 platinum-resistant recurrent |  | Platinum | CNVs detected through OncoScan (involving 9 genes), SNPs of OncoScan for the calculating the HR score (including LOH, LST, tAI, CS), mutational profile of 24-gene panel, | 2 | D | 2 | 2 | 3 |
| Via_reviews | High EMSY expression defines a BRCA-like subgroup of high-grade serous ovarian carcinoma with prolonged survival and hypersensitivity to platinum |  |  | Cancer. 2019 Aug 15;125(16):2772-2781. | Prospective study, but EMSY retrospectively analyzed | Newly diagnosed HGSC |  | Platinum+Bev | All patients evaluated for RNA expression, especially EMSY, and validating the EMSY cut-off in another cohort | 1 | B | 1 | 1 | 3 |
| Via_reviews | Overall survival and updated progression-free survival outcomes in a randomized phase II study of combination cediranib and olaparib versus olaparib in relapsed platinum-sensitive ovarian cancer | NCT0111648 |  | Ann Oncol. 2019 Apr 1;30(4):551-557. | Phase II, two arm | 90 Platinum-sensitive recurrent, high-grade serous or endometrioid | Salvage, 1:1 (cediranib+olaparib:olaparib) | Olaparib with or without cediranib | All enrolled patients having known germline BRCA1/2 status | 1 | B | 1 | 1 | 3 |
| Via_reviews | Genetic and epigenetic profiling of BRCA1/2 in ovarian tumours reveals additive diagnostic yield and evidence of a genomic BRCA1/2 DNA methylation signature |  |  | J Hum Genet. 2020 Oct;65(10):865-873. | Retrospective | EOC, HGSC |  | NA | Using NGS to detect SNP, CNV and DNA methylation within BRCA1/2 genes; might be helpful when discussing DNA methylation | 1 | D | 2 | 2 | 3 |
| Via_reviews | The RECAP Test Rapidly and Reliably Identifies Homologous Recombination-Deficient Ovarian Carcinomas |  |  | Cancers (Basel). 2020 Sep 29;12(10):2805 | Retrospective | Newly diagnosed EOC | Frontline | Platinum-based chemotherapy | NGS for BRCA1/2, variants of HRR-related genes and LOH; BRCA1 promoter methylation; RECAP (RAD51 functional assay) | 3 | D | 2 | 2 | 3 |
| Via_reviews | SLFN11 captures cancer-immunity interactions associated with platinum sensitivity in high-grade serous ovarian cancer |  |  | JCI Insight. 2021 Sep 22;6(18):e146098. | Preclinical study | Cells from HGSC |  | Cisplatin | Stained for intracellular SLFN11, analysed by flowmetry | 4 | NA (preclinical study) | 2 | 2 | 3 |
| Via_reviews | A RAD51 functional assay as a candidate test for homologous recombination deficiency in ovarian cancer | NCT01583322 | CHIVA | Gynecol Oncol. 2023 Apr;171:106-113. | Phase 2 with archival samples used | Newly diagnosed stage III-IV, for NACT | Neoadjuvant | Carboplatin-paclitaxel | Immunofluorescence for nuclear RAD51, geminin, γH2AX and DAPI; some also evaluated by 411-cancer-gene panel | 3 | A | 1 | 1 | 2 |

HRD: Homologous Recombination Deficiency; LOE: Level of evidence; EGAPP: Evaluation of Genomic Applications in Practice and Prevention

### Appendix Table 3: Details of Studies Included in the Meta-Analyses on the Efficacy of Predictive Biomarkers of PARP Inhibitors in (A) the Frontline Setting and (B) the Recurrent Setting

| 1. **Details of studies included in the meta-analysis on the efficacy of predictive biomarkers of PARP inhibitors in the frontline setting** | | | | | | | | | | | | |
| --- | --- | --- | --- | --- | --- | --- | --- | --- | --- | --- | --- | --- |
| **Study** | **Participants** | **Sample_size** | **Biomarker** | **Drug** | **TE** | **seTE** | **95% LCI** | **95%UCI** | **PFS (PARPi)** | **PFS (Control)** | **P** | **Reference** |
| DiSilvestro et al._2023 (SOLO1) | Stage III-IV, high-grade serous or endometrioid | 391 | g/t BRCA1/2 | olaparib vs placebo | 0.55 | 0.3312 | 0.4 | 0.76 | 49.9 | 13.8 | <0.001 | J Clin Oncol. 2023 Jan 20;41(3):609-617. |
| Banerjee et al._2021 (SOLO1) | Stage III-IV, high-grade serous or endometrioid | 391 | g/t BRCA1/2 | olaparib vs placebo | 0.33 | 0.1395 | 0.25 | 0.43 | 56 | 13.8 | <0.001 | Lancet Oncol. 2021 Dec;22(12):1721-1731. |
| Coleman et al._2019 (VELIA) | Stage III-IV, HGSC | 1140 | g/t BRCA1/2 | veliparib vs placebo | 0.44 | 0.2262 | 0.28 | 0.68 | 34.7 | 22 | <0.001 | N Engl J Med. 2019 Dec 19;381(25): 2403-2415 |
| Coleman et al._2019 (VELIA) | Stage III-IV, HGSC | 1140 | HRD (Myriad myChoice, cut-off 33) | veliparib vs placebo | 0.57 | 0.1439 | 0.43 | 0.76 | 31.9 | 20.5 | <0.001 | N Engl J Med. 2019 Dec 19;381(25): 2403-2415 |
| Ray-Coquard et al._2023 (PAOLA-1) | Stage III-IV, high-grade serous or endometrioid | 806 | t BRCA1/2 | olaparib+bev vs placebo+bev | 0.45 | 0.1767 | 0.32 | 0.64 | 60.7 | 21.7 | <0.001 | Ann Oncol. 2023 Aug;34(8):681-692 |
| Ray-Coquard et al._2023 (PAOLA-1) | Stage III-IV, high-grade serous or endometrioid | 806 | HRD (Myriad myChoice, cut-off 42) | olaparib+bev vs placebo+bev | 0.41 | 0.1341 | 0.32 | 0.54 | 46.8 | 17.6 | <0.001 | Ann Oncol. 2023 Aug;34(8):681-692 |
| González-Martín et al._2023 (PRIMA) | Stage III-IV, high-grade serous or endometrioid | 733 | HRD (Myriad myChoice, cut-off 42) | niraparib vs placebo | 0.52 | 0.1353 | 0.4 | 0.68 | 24.5 | 11.2 | <0.001 | Eur J Cancer. 2023 Aug:189:112908. |
| Monk et al._2022 (ATHENA-MONO) | Stage III-IV, HGSC | 538 | HRD (FiundationOne) | Rucaparib vs Placebo | 0.47 | 0.2149 | 0.31 | 0.72 | 28.7 | 11.3 | 0.0004 | J Clin Oncol. 2022 Dec 1;40(34):3952-3964. |
| 1. **Details of studies included in the meta-analysis on the efficacy of predictive biomarkers of PARP inhibitors in the recurrent setting** | | | | | | | | | | | | |
| **Study** | **Participants** | **Sample_size** | **Biomarker** | **Drug** | **TE** | **seTE** | **95% LCI** | **95% UCI** | **PFS (PARPi)** | **PFS (Control)** | **P** | **Reference** |
| Ledermann et al._2014 (Study 19, phase 2) | Platinum-sensitive, HGSC | 265 | g/t BRCA1/2 | Olaparib vs Placebo | 0.22 | 0.307 | 0.12 | 0.4 | 11.2 | 4.3 | <0.0001 | Lancet Oncol. 2014 Jul;15(8):852-61. |
| Del Campo et al._2019 (NOVA) | Platinum-sensitive | 553 | g BRCA1/2 | Niraparib vs Placebo | 0.24 | 0.31 | 0.131 | 0.441 |  |  | <0.0001 | J Clin Oncol. 2019 Nov 10;37(32):2968-2973. |
| Swisher et al._2017 (ARIEL2) | Platinum-sensitive, high-grade serous or endometrioid | 256 | t BRCA1/2 | Rucaparib vs Placebo | 0.27 | 0.2583 | 0.16 | 0.44 |  |  | <0.0001 | Lancet Oncol. 2017 Jan;18(1):75-87. |
| Swisher et al._2017 (ARIEL2) | Platinum-sensitive, high-grade serous or endometrioid | 256 | HRD (FoundationOne) | Rucaparib vs Placebo | 0.62 | 0.195 | 0.42 | 0.9 |  |  | 0.011 | Lancet Oncol. 2017 Jan;18(1):75-87. |
| Coleman et al._2017 (ARIEL3) | Platinum-sensitive, high-grade serous or endometrioid | 564 | t BRCA1/2 | Rucaparib vs Placebo | 0.23 | 0.1922 | 0.16 | 0.34 | 16.6 | 5.4 | <0.0001 | Lancet. 2017 Oct 28;390(10106):1949-1961. |
| Coleman et al._2017 (ARIEL3) | Platinum-sensitive, high-grade serous or endometrioid | 564 | HRD (FoundationOne) | Rucaparib vs Placebo | 0.32 | 0.1429 | 0.24 | 0.42 | 13.6 | 5.4 | <0.0001 | Lancet. 2017 Oct 28;390(10106):1949-1961. |
| Pujade-Lauraine et al._2017 (SOLO2) | Platinum-sensitive | 295 | g/t BRCA1/2 | Olaparib vs Placebo | 0.3 | 0.1586 | 0.22 | 0.41 | 19.1 | 5.5 | <0.0001 | Lancet Oncol. 2017 Sep;18(9):1274-1284. |
| Oza et al._2015 (Phase 2) | Platinum-sensitive, HGSC | 41 | BRCA1/2 | Olaparib + Chemotherapy vs Chemotherapy alone | 0.21 | 0.4915 | 0.08 | 0.55 |  |  | 0.0015 | Lancet Oncol. 2015 Jan;16(1):87-97. |
| Penson et al._2020 (SOLO3) | Platinum-sensitive, high-grade serous or endometrioid | 266 | g BRCA1/2 | Olaparib vs single non-platinum chemotherapy | 0.62 | 0.1914 | 0.43 | 0.91 | 13.4 | 9.2 | 0.013 | J Clin Oncol. 2020 Apr 10;38(11):1164-1174 |
| Wu et al._ 2021 (NORA) | Platinum-sensitive | 265 | g BRCA1/2 | Niraparib vs Placebo | 0.22 | 0.3009 | 0.12 | 0.39 | NR | 5.5 | <0.0001 | Ann Oncol. 2021 Apr;32(4):512-521. |
| Kristeleit et al._2022 (ARIEL4) | Platinum-sensitive and platinum-resistant | 325 | g/s BRCA1/2 | Rucaparib vs Chemotherapy | 0.64 | 0.1376 | 0.49 | 0.84 | 7.4 | 5.7 | 0.001 | Lancet Oncol. 2022 Apr;23(4):465-478. |

### Appendix Table 4: Summary of key findings from systematic review by category of biomarkers

| 1. **Studies utilizing *BRCA1/2* pathogenic variants to predict PARP inhibitors efficacy** | | | | |
| --- | --- | --- | --- | --- |
| **Study** | **Study type** | **Reference** | **Biomarker** | **Main finding** |
| VELIA (NCT02470585) | Phase 3 | Coleman et al. N Engl J Med. 2019 Dec 19;381(25): 2403-2415; [12] | g/s BRCA1/2 | Original result of VELIA: adding veliparib to chemotherapy followed by maintenance therapy improves PFS in *BRCA1/2*-mutated or HRD-positive ovarian cancer |
|  |  | Aghajanian et al. Gynecol Oncol. 2022 Feb;164(2):278-287 | g/s BRCA1/2 | Post-hoc analysis of VELIA: weekly paclitaxel contributing to longer PFS in HR proficient and *BRCA1/2* wild-type patients |
|  |  | Swisher et al. Gynecol Oncol. 2022 Feb;164(2):245-253 | g/s BRCA1/2 | Post-hoc analysis of VELIA: patients with *BRCA1/2* wild-type and HR proficiency also benefit from veliparib |
|  |  | Mizuno et al. Int J Clin Oncol. 2023 Jan;28(1):163-174 | g/s BRCA1/2 | Subgroup analysis of VELIA: Japanese cohort, consistent with the overall trial findings |
| NOVA (NCT01847274) | Phase 3 | Campo et al. J Clin Oncol. 2019 Nov 10;37(32):2968-2973. | g BRCA1/2 | Post-hoc analysis of NOVA: niraparib maintenance prolonged PFS in platinum-sensitive, recurrent ovarian cancer, regardless of their response to the last platinum and status of germline *BRCA1/2* |
| PRIMA (NCT02655016) | Phase 3 | González-Martín et al. N Engl J Med. 2019 Dec 19;381(25):2391-2402; [13] | t BRCA1/2 | Original result of PRIMA: niraparib improves PFS in newly diagnosed ovarian cancer who had responded to chemotherapy, regardless of HRD status |
|  |  | González-Martín et al. Eur J Cancer. 2023 Aug;189:112908; [21] | t BRCA1/2 | Updated PFS of the PRIMA trial |
| PAOLA-1 (NCT02477644) | Phase 3 | Ray-Coquard et al. N Engl J Med. 2019 Dec 19;381(25):2416-2428; [14] | t BRCA1/2 | Original result of PAOLA-1: olaparib plus bevacizumab as maintenance in ovarian cancer improves PFS, especially in those with tumour HRD |
|  |  | Fujiwara et al. J Gynecol Oncol. 2021 Sep;32(5):e82 | t BRCA1/2 | Subgroup analysis of PAOLA-1: Japanese cohort, consistent with the overall trial findings |
|  |  | Pujade-Lauraine et al. JCO Precis Oncol. 2023 Jan;7:e2200258; [51] | t BRCA1/2 | Post-hoc analysis of PAOLA-1: non-*BRCA1/2* gene panels failed to predict olaparib plus bevacizumab benefit in ovarian cancer |
|  |  | Ray-Coquard et al. Ann Oncol. 2023 Aug;34(8):681-692; [20] | t BRCA1/2 | Updated result of PAOLA-1: maintenance olaparib plus bevacizumab do not improve OS in the overall population but do show OS improvement in HRD patients |
|  |  | Callens et al. J Natl Cancer Inst. 2021 Jul 1;113(7):917-923 | t BRCA1/2 | Subgroup analysis of PAOLA-1: French cohort, consistent with the overall trial findings; t*BRCA1/2* and g*BRCA1/2* showed compatible predictability in the efficacy of PARP inhibitor |
| SOLO3 (NCT02282020) | Phase 3 | Penson et al. J Clin Oncol. 2020 Apr 10;38(11):1164-1174; [24] | g BRCA1/2 | Original result of SOLO3: Olaparib showed survival benefits compared to nonplatinum chemotherapy in germline *BRCA1/2*-mutated platinum-sensitive recurrent ovarian cancer |
| SOLO1 (NCT01844986) | Phase 3 | Banerjee et al. Lancet Oncol. 2021 Dec;22(12):1721-1731; [23] | g/t BRCA1/2 | Updated result of SOLO1: olaparib extended PFS in newly diagnosed advanced ovarian cancer patients with *BRCA1/2* pathogenic variants |
|  |  | Wu et al. Gynecol Oncol. 2021 Jan;160(1):175-181 | g/t BRCA1/2 | Subgroup analysis of SOLO1: Chinese cohort, consistent with the overall trial findings |
|  |  | DiSilvestro et al. J Clin Oncol. 2023 Jan 20;41(3):609-617 | g/t BRCA1/2 | Updated result of SOLO1: maintenance olaparib improved OS in advanced ovarian cancer with *BRCA1/2* pathogenic variants |
| ARIEL3 (NCT01968213) | Phase 3 | Clamp et al. Int J Gynecol Cancer. 2021 Jul;31(7):949-958 | g/t BRCA1/2 | Post-hoc analysis of ARIEL3: rucaparib improved PFS in recurrent ovarian cancer across various subgroups, including platinum sensitivity |
|  |  | Oaknin et al. Cancer Med. 2021 Oct;10(20):7162-7173 | g/t BRCA1/2 | Rucaparib maintenance showed PFS benefits, regardless of previous platinum sensitivity or disease burden |
|  |  | O'Malley et al. Gynecol Oncol. 2022 Dec;167(3):404-413; [48] | g/t BRCA1/2 | Post-hoc analysis of ARIEL3: rucaparib maintenance showed benefit in recurrent ovarian cancer, particularly for those with characteristics related to HRD |
| NORA (NCT03705156) | Phase 3 | Wu et al. Ann Oncol. 2021 Apr;32(4):512-521; [77] | g BRCA1/2 | Original result of NORA: niraparib maintenance extended PFS in platinum-sensitive recurrent ovarian cancer |
| ARIEL4 (NCT02855944) | Phase 3 | Kristeleit et al. Lancet Oncol. 2022 Apr;23(4):465-478; [78] | g/s BRCA1/2 | Original result of ARIEL4: rucaparib as an alternative to chemotherapy for recurrent ovarian cancer with *BRCA1/2* pathogenic variants |
| NRG-GY004 (NCT02446600) | Phase 3 | Liu et al. J Clin Oncol. 2022 Jul 1;40(19):2138-2147. | g BRCA1/2 | Original results of NRG-GY004: olaparib/cediranib not improve PFS in platinum-sensitive recurrent ovarian cancer compared to chemotherapy but showed benefits in *BRCA1/2*-mutated patients |
| ATHENA-MONO (NCT03522246) | Phase 3 | Monk et al. J Clin Oncol. 2022 Dec 1;40(34):3952-3964; [64] | t BRCA1/2 | Original result of ATHENA-MONO: rucaparib maintenance showed benefit for advanced ovarian cancer with and without HRD |
| Study 10 and ARIEL2 (NCT01482715, NCT01891344) | Phase 2 | Kristeleit et al. Int J Gynecol Cancer. 2019 Nov;29(9):1396-1404. | t BRCA1/2 | Post-hoc analysis of study 10 and ARIEL2: rucaparib showing antitumor activity for platinum-sensitive, *BRCA1/2*-mutated ovarian cancer |
|  |  | Kristeleit et al. Br J Cancer. 2023 Jan;128(2):255-265. | t BRCA1/2 | Updated result of study 10: rucaparib showed antitumor activity in heavily pretreated ovarian cancer patients with *BRCA1/2* pathogenic variants. |
|  |  | Liu et al. Asia Pac J Clin Oncol. 2022 Dec;18(6):714-722 | g/t BRCA1/2 | Subgroup analysis of SOLO2: Chinese cohort, consistent with the overall trial findings |
| LIGHT (NCT02983799) | Phase 2 | Cadoo et al. Gynecol Oncol. 2022 Sep;166(3):425-431; [39] | g/t BRCA1/2 | Original result of LIGHT: olaparib showed survival benefits for platinum-sensitive recurrent ovarian cancer, especially in *BRCA1/2* mutated and HRD-positive tumours |
| OVARIO (NCT03326193) | Phase 2 | Hardesty et al. Gynecol Oncol. 2022 Aug;166(2):219-229; [40] | t BRCA1/2 | Original result of OVARIO: niraparib plus bevacizumab maintenance showed promising PFS in newly-diagnosed ovarian cancer |
| NCT0111648 | Phase 2 | Liu et al. Ann Oncol. 2019 Apr 1;30(4):551-557. | g BRCA1/2 | Cediranib/olaparib extended PFS in relapsed platinum-sensitive ovarian cancer, particularly for those without germline *BRCA1/2* pathogenic variants |
|  | Retrospective | Agarwal et al. JCO Glob Oncol. 2021 Apr;7:506-511 | g BRCA1/2 | PARP inhibitors demonstrated promise in platinum-resistant ovarian cancer with germline *BRCA1/2* pathogenic variants (single-centre experience) |
| 1. **Studies utilizing HRR-related genes beyond BRCA1/2 to predict PARP inhibitors efficacy** | | | | |
| **Study** | **Study type** | **Reference** | **Biomarker** | **Main finding** |
| PAOLA-1 (NCT02477644) | Phase 3 | Pujade-Lauraine et al. JCO Precis Oncol. 2023 Jan;7:e2200258; [51] | non-BRCA1/2 HRR-related gene panels | Post-hoc analysis of PAOLA-1: pathogenic variants in non-*BRCA1/2* HRD gene did not predict survival benefit with maintenance olaparib plus bevacizumab in ovarian cancer |
| Study 10 and ARIEL2 (NCT01482715, NCT01891344) | Phase 2 | Kristeleit et al. Int J Gynecol Cancer. 2019 Nov;29(9):1396-1404. | BROCA assay (including ≥ 15 HRR-related genes) | Post-hoc analysis of study 10 and ARIEL2: rucaparib showed effectiveness for platinum-sensitive, *BRCA1/*2-mutated ovarian cancer |
|  | Phase 2 | Swisher et al. Nat Commun. 2021 May 3;12(1):2487; [47] | BROCA assay (including ≥ 15 HRR-related genes) | Post-hoc analysis of ARIEL2: pathogenic variants of *RAD51C/D* and *BRCA1* promoter methylation predict rucaparib response in recurrent ovarian cancer |
| AMBITION (NCT03699449) | Phase 2 | Lee et al. J Gynecol Oncol. 2022 Jul;33(4):e45; [50] | HRR-gene panel | Original result of AMBITION: biomarker-driven combination therapies showed promise for heavily pre-treated platinum-resistant ovarian cancer patients |
|  | Retrospective | Hollis et al. Cancer. 2019 Aug 15;125(16):2772-2781; [45] | EMSY | High EMSY expression in ovarian cancer also linked to increased platinum sensitivity, and better response to PARP inhibitors |
|  | Preclinical study | Coelho et al. Cell Death Dis. 2022 Oct 28;13(10):909. | CRISPR-Cas9 (evaluating 93 candidate genes) | CRISPR-Cas9 mutagenesis screening identified predictive markers (*ATM, MUS81, NBN, BRCA2, RAD51B*) for olaparib response in ovarian cancer |
|  | Preclinical study | Gundogdu et al. Cell Signal. 2021 Nov;87:110106; [43] | hMOB2 | *hMOB2* regulates double-strand break repair and supports cancer cell survival, making it a potential biomarker for PARP inhibitors. |
|  | Preclinical study | Morra et al. J Exp Clin Cancer Res. 2022 Aug 13;41(1):245; [44] | CCDC6-PP4 | CCDC6 loss enhances PP4c complex activity, causing PARP inhibitor sensitivity in ovarian cancer cells |
| 1. **Studies utilizing methylation of HRR-related genes to predict PARP inhibitors efficacy** | | | | |
| **Study** | **Study type** | **Reference** | **Biomarker** | **Main finding** |
| Study 10 and ARIEL2 (NCT01482715, NCT01891344) | Phase 2 | Swisher et al. Nat Commun. 2021 May 3;12(1):2487; [47] | Promoter methylation of *RAD51C* and *BRCA1* | Post-hoc analysis of ARIEL2: *RAD51C/D* pathogenic variants and *BRCA1* promoter methylation predict rucaparib response in ovarian carcinoma |
|  |  | Kristeleit et al. Int J Gynecol Cancer. 2019 Nov;29(9):1396-1404. | Promoter methylation of *RAD51C* and *BRCA1* | Post-hoc analysis of study 10 and ARIEL2: various biomarkers analysed for predicting rucaparib’s efficacy in *BRCA1/2*-mutated ovarian cancer; limited predictability of *BRCA1* and *RAD51C* promoter methylation |
| TOPACIO | Phase 2 | Färkkilä et al. Nat Commun. 2020 Mar 19;11(1):1459; [49] | *BRCA1* promoter methylation | Original result of TOPACIO: various biomarkers analysed for the predictability for the efficacy of niraparib and pembrolizumab; limited predictability of *BRCA1* promoter methylation |
|  | Retrospective | Sahnane et al. Int J Mol Sci. 2020 Dec 19;21(24):9708; [56] | *BRCA1/2* promoter methylation | A portion of ovarian cancer without *BRCA1/2* pathogenic variants exhibit *BRCA1/2* promoter methylation, suggesting potential responsiveness to PARP inhibitors |
|  | Retrospective | Aref-Eshghi et al. J Hum Genet. 2020 Oct;65(10):865-873; [55] | Promoter methylation of *BRCA1/2* | Combining DNA analysis techniques can diagnose additional 10% BRCAness by identifying methylation and CNV within *BRCA1/2* in ovarian tumours |
|  | Preclinical study | Pellegrino et al. Cancer Res. 2022 Apr 15;82(8):1646-1657; [57] | *BRCA1* promoter methylation | Various biomarkers analysed for predicting the efficacy of PARP inhibitors and results showed limited predictability of *BRCA1* promoter methylation |
|  | Preclinical study | Guffanti et al. Br J Cancer. 2022 Jan;126(1):120-128; [34] | *BRCA1* promoter methylation | Various biomarkers analysed for predicting the efficacy of PARP inhibitors and results showed limited predictability of *BRCA1* promoter methylation |
| 1. **Studies utilizing genomic scars or mutational signature to predict PARP inhibitors efficacy** | | | | |
| **Study** | **Study type** | **Reference** | **Biomarker** | **Main finding** |
| VELIA (NCT02470585) | Phase 3 | Coleman et al. N Engl J Med. 2019 Dec 19;381(25): 2403-2415; [12] | Genomic scars (Myriad myChoice, 33) | Original result of VELIA: adding veliparib to chemotherapy followed by maintenance therapy improves PFS in *BRCA1/2*-mutated or HRD-positive ovarian cancer |
|  |  | Aghajanian et al. Gynecol Oncol. 2022 Feb;164(2):278-287 |  | Post-hoc analysis of VELIA: weekly paclitaxel contributing to longer PFS in HR proficient and *BRCA1/2* wild-type patients |
|  |  | Swisher et al. Gynecol Oncol. 2022 Feb;164(2):245-253 |  | Post-hoc analysis of VELIA: *BRCA1/2* wild-type and HR proficiency ovarian cancer also benefit from veliparib |
| PRIMA (NCT02655016) | Phase 3 | González-Martín et al. N Engl J Med. 2019 Dec 19;381(25):2391-2402; [13] | Genomic scars (Myriad myChoice, 42) | Original result of PRIMA: niraparib improves PFS in newly diagnosed ovarian cancer who had responded to chemotherapy, regardless of HRD status |
|  |  | González-Martín et al. Eur J Cancer. 2023 Aug;189:112908; [21] |  | Updated PFS of the PRIMA trial. |
| PAOLA-1 (NCT02477644) | Phase 3 | Ray-Coquard et al. N Engl J Med. 2019 Dec 19;381(25):2416-2428; [14] | Genomic scars (Myriad myChoice, 42) | Original result of PAOLA-1: olaparib plus bevacizumab as maintenance improves PFS in patients with ovarian cancer, especially in those with tumour HRD |
|  |  | Fujiwara et al. J Gynecol Oncol. 2021 Sep;32(5):e82 |  | Subgroup analysis of PAOLA-1: Japanese cohort, consistent with the overall trial findings |
|  |  | Callens et al. J Natl Cancer Inst. 2021 Jul 1;113(7):917-923 |  | Subgroup analysis of PAOLA-1: French cohort, consistent with the overall trial findings |
|  |  | Harter et al. Gynecol Oncol. 2022 Feb;164(2):254-264 |  | Post-hoc analysis of PAOLA-1: olaparib plus bevacizumab improves PFS in higher-risk and lower-risk patients, particularly in HRD-positive patients, based on analysis of clinical risks and HRD status |
|  |  | Ray-Coquard et al. Ann Oncol. 2023 Aug;34(8):681-692; [20] |  | Updated result of PAOLA-1: maintenance olaparib plus bevacizumab not improve OS in the overall population but do show OS improvement in HRD patients |
|  |  | Loverix et al. Eur J Cancer. 2023 Jul;188:131-139. | Genomic scars (Myriad myChoice vs Leuven assay) | The Leuven HRD test correlates well with the Myriad HRD test in predicting the HRD status in the PAOLA-1 trial. |
|  |  | Christinat et al. JCO Precis Oncol. 2023 Jun;7:e2200555. | Genomic scars (Myriad myChoice vs Geneva assay) | The Geneva assay correlates well with the Myriad HRD test in predicting the HRD status in the PAOLA-1 trial with a lower failure rate. |
| ARIEL3 (NCT01968213) | Phase 3 | Clamp et al. Int J Gynecol Cancer. 2021 Jul;31(7):949-958 | Genomic scars (FoundationOne, 16%) | Post-hoc analysis of ARIEL3: rucaparib maintenance improves PFS for recurrent ovarian cancer, regardless of number of prior chemotherapies or prior use of bevacizumab |
|  |  | Oaknin et al. Cancer Med. 2021 Oct;10(20):7162-7173 |  | Post-hoc analysis of ARIEL3: rucaparib maintenance improves PFS in recurrent ovarian cancer, regardless of recent platinum sensitivity or disease burden |
|  |  | O'Malley et al. Gynecol Oncol. 2022 Dec;167(3):404-413; [48] |  | Post-hoc analysis of ARIEL3: rucaparib maintenance showed better PFS in recurrent ovarian cancer, particularly among those with characteristics of HRD |
| OPINION (NCT03402841) | Phase 3 | Poveda et al. Gynecol Oncol. 2022 Mar;164(3):498-504; [62] | Genomic scars (Myriad myChoice, 42) | Original result of OPINION: olaparib maintenance benefits in platinum-sensitive relapsed ovarian cancer without germline *BRCA1/2* pathogenic variants, with varying PFS based on biomarkers |
| ATHENA-MONO (NCT03522246) | Phase 3 | Monk et al. J Clin Oncol. 2022 Dec 1;40(34):3952-3964; [64] | Genomic scars (FoundationOne, 16%) | Original result of ATHENA-MONO: rucaparib maintenance demonstrated efficacy for advanced ovarian cancer regardless of HRD status |
| MITO16A (NCT01706120) | Phase 4 | Capoluongo et al. ESMO Open. 2022 Oct;7(5):100585; [63] | Genomic scars (Myriad myChoice, 42 and Agilent OneSeq Panel) | Post-hoc analysis of MITO16A: academic genomic tests show high sensitivity and concordance with MyChoice CDx in detecting HRD in ovarian cancer |
| QUADRA (NCT03759600) | Phase 2 | Okamoto et al. J Gynecol Oncol. 2021 Mar;32(2):e16 | Genomic scars (Myriad myChoice, 42) | Subgroup analysis of QUADRA: Japanese cohort, consistent with the overall trial findings |
| LIGHT (NCT02983799) | Phase 2 | Cadoo et al. Gynecol Oncol. 2022 Sep;166(3):425-431; [39] | Genomic scars (Myriad myChoice, 42) | Original result of LIGHT: Olaparib showed effectiveness in platinum-sensitive relapsed ovarian cancer, especially in those with *BRCA1/2* pathogenic variants and HRD |
| OVARIO (NCT03326193) | Phase 2 | Hardesty et al. Gynecol Oncol. 2022 Aug;166(2):219-229; [40] | Genomic scars (Myriad myChoice, 42) | Original result of OVARIO: Niraparib plus bevacizumab as maintenance in advanced ovarian cancer showed promising PFS |
| TOPACIO (NCT02657889) | Phase 2 | Färkkilä et al. Nat Commun. 2020 Mar 19;11(1):1459; [49] | Mutational signature 3 | Original result of TOPACIO: response to niraparib and pembrolizumab in ovarian cancer best determined by mutational signature 3 and a positive immune score |
|  | Retrospective | Wen et al. BMC Cancer. 2022 May 16;22(1):550. | Genomic scar (GeneseeqPrime HRD, 38) | HRD determined by the GeneseeqPrime assay predict the effectiveness of platinum in patients without *BRCA1/2* pathogenic variants |
|  | Retrospective | da Costa et al. BMC Cancer. 2019 May 6;19(1):422; [61] | Genomic scar (OncoScan) | Ovarian cancer with prolonged platinum sensitivity and HRD respond better to platinum retreatment, especially if they lack *CCNE1* gains or have *RB1* loss |
|  | Preclinical study | Guffanti et al. Br J Cancer. 2022 Jan;126(1):120-128; [34] | HRDetect | Tumor HRD determined by RAD51 but not HRDetect, serves as a potential predictive biomarker for olaparib response in ovarian cancer |
|  | Preclinical study | Pellegrino et al. Cancer Res. 2022 Apr 15;82(8):1646-1657; [57] | Genomic scars (Myriad myChoice, 42) and HRDetect | HRDetect demonstrates higher sensitivity but low specificity in predicting the response to PARP inhibitors and in various cancer types |
| 1. **Studies utilizing RAD51 functional assay to predict PARP inhibitors efficacy** | | | | |
| **Study** | **Study type** | **Reference** | **Biomarker** | **Main finding** |
| TOPACIO (NCT02657889) | Phase 2 | Färkkilä et al. Nat Commun. 2020 Mar 19;11(1):1459; [49] | IHC of RAD51 | Various biomarkers analysed, only mutational signature 3 and a positive immune score, provided insight into predictive biomarkers for the combination of pembrolizumab and niraparib |
| CHIVA (NCT01583322) | Phase 2 | Blanc-Durand et al. Gynecol Oncol. 2023 Apr;171:106-113; [73] | Immunofluorescence of RAD51 | Post-hoc analysis of CHIA: RAD51-low tumours showing increased platinum sensitivity |
| MITO16A (NCT01706120) | Phase 4 | Capoluongo et al. ESMO Open. 2022 Oct;7(5):100585; [63] | Immunofluorescence of RAD51 | Post-hoc analysis of MITO16A: genomic and functional tests for HRD showed good agreement with Myriad |
|  | Retrospective | van Wijk et al. Cancers (Basel). 2020 Sep 29;12(10):2805; [72] | RECAP (RAD51 assay) | The RAD51 foci-based RECAP test effectively identifies HRD, and RECAP-positive associating with better survival with platinum |
|  | Case series | Tao et al. Pharmacol Res. 2022 May;179:106232.; [35] | Immunofluorescence of RAD51 | Patient-derived organoids can effectively assess the sensitivity to PARP inhibitors and platinum |
|  | Preclinical study | Guffanti et al. Br J Cancer. 2022 Jan;126(1):120-128; [34] | Immunofluorescence of RAD51 | Low basal RAD51 foci scores in ovarian cancer patient-derived xenografts predict better sensitivity to olaparib and platinum therapy |
|  | Preclinical study | Pellegrino et al. Cancer Res. 2022 Apr 15;82(8):1646-1657; [57] | Immunofluorescence of RAD51 | RAD51 nuclear foci predicts the response to PARP inhibitors and platinum in ovarian cancer |
| 1. **Studies utilizing other biomarkers to predict PARP inhibitors efficacy** | | | | |
| **Study** | **Study type** | **Reference** | **Biomarker** | **Main finding** |
| Study 10 and ARIEL2 (NCT01482715, NCT01891344) | Phase 2 | Colomban et al. EBioMedicine. 2023 Mar;89:104477; [36] | KELIM-PARP | Post-hoc analysis of Study 10 and ARIEL2: mathematical modelling of CA-125 levels during rucaparib treatment can predict its efficacy, particularly for platinum-sensitive *BRCA1/2*-mutated ovarian cancer |
|  | Retrospective | Conrad et al. Mol Cancer Ther. 2020 Jan;19(1):282-291; [30] | ADP-Ribosylation | ADP-ribosylation levels in ovarian cancers could be a biomarker for predicting responses to chemotherapy and PARP inhibitors |
| NCT01472783 | Phase 2 | Rusan et al. Eur J Cancer. 2020 Jan;125:121-129; [31] | HOXA9 promoter methylation | HOXA9 methylation in circulating tumour DNA predicts clinical outcomes in platinum-resistant *BRCA1/2*-mutated ovarian cancer treated with PARP inhibitors |
|  | Preclinical study | Sheta et al. J Transl Med. 2020 Nov 19;18(1):439; [32] | Patient-derived organoids | 3D patient-derived organoids indicate PARP inhibitor sensitivity in ovarian cancer |
|  | Case series | Tao et al. Pharmacol Res. 2022 May;179:106232;[35] |  | Patient-derived organoids can effectively assess the sensitivity to PARP inhibitors and platinum |
|  | Preclinical study | Winkler et al. JCI Insight. 2021 Sep 22;6(18):e146098; [33] | SLFN11 | SLFN11 levels in ovarian cancer predict better outcomes and immune activation during platinum-based chemotherapy. |
